# Copper nanoparticles anchored onto boron-doped graphene nanosheets for use as a high performance asymmetric solid-state supercapacitor†

**DOI:** 10.1039/c8ra08762h

**Published:** 2019-01-25

**Authors:** P. Muthu Pandian, A. Pandurangan

**Affiliations:** Department of Chemistry, Anna University Chennai 600 025 Tamil Nadu India pandurangan_a@yahoo.com +91 44 22200660 +91 44 22358653

## Abstract

There is a high demand for high energy and power density in the field of energy storage devices. To rectify these limitations, a novel asymmetric solid-state supercapacitor (ASSC) was designed and fabricated using a copper anchored boron doped graphene nanosheet (CuBG) as a negative electrode and reduced graphene nanoplatelets as a positive electrode with H_2_SO_4_/PVA as the quasi-solid electrolyte. The CuBG was prepared using a two step hydrothermal process followed by pyrolysis at different temperatures using chemical vapour deposition (CVD), using copper sulphate (CuSO_4_) and boron-trioxide (B_2_O_3_) as precursors, for doping in graphene oxide. Owing to the remarkable structure and morphology of Cu nanoparticles on nanosheets of boron intercalated with graphene oxide, the nanosheets exhibit a high specific capacitance of 483 Fg^−1^ at 1 Ag^−1^ with a capacitance retention of 96% after 5000 cycles, respectively, in a two-electrode system. In addition, the designed and fabricated solid state ASSC device of rGO//CuBG exhibited a high energy and power density of 132.5 W h kg^−1^ and 1000 W kg^−1^, respectively, in a wide potential window of 2.0 V, with an excellent stability, retaining 91% of its initial specific capacitance after 5000 cycles. The electrochemical capacitance of CuBG was also evaluated in a three and two electrode system using a KOH and KOH/PVA solid electrolyte respectively. A specific capacitance of 87.5 Fg^−1^ was achieved at 1 Ag^−1^ using the fabricated asymmetric device with a 31.1 W h kg^−1^ energy density at a corresponding power density of 800 W kg^−1^ and an 85% capacitance was retained after 5000 cycles. The kinetics of the interfacial charge transport phenomena were analysed using a Nyquist plot of the electrochemical impedance analysis.

## Abstract 2

There is a high demand for high energy and power density in the field of energy storage devices.

## Graphical abstract

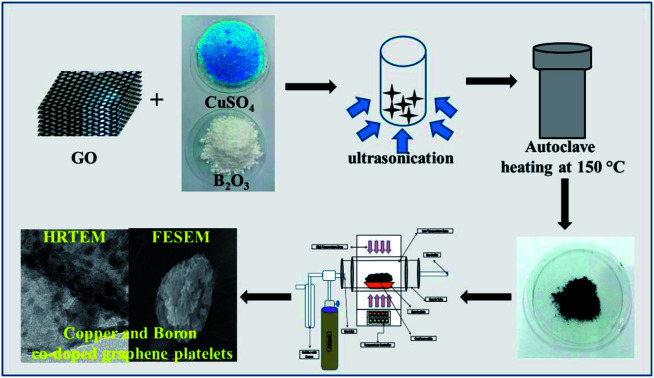

## Introduction

Commercially available supercapacitors still abide by low energy densities which are incapable of being exchanged for a battery as a power source.^1,2^ Pseudo capacitors and electrical double layer capacitors (EDLCs) are the two categories of supercapacitor electrode materials owing to their fast reduction, oxidation and reversible reaction pseudocapacitance that can produce a higher capacitance and energy density than EDLCs.^3–5^ Owing to the presence of the functional groups containing oxygen, graphene demonstrates a higher capacitance. To increase the energy density and potential window of the working device, electrode materials play a vital role in a supercapacitor.^6^ As the alloys and noble metals are very costly and have poisoning effects,^7^ low-cost nanostructures of transition metal oxides, such as NiO,^3^ CuO,^8^ Fe_2_O_3_, Gd^9^ and Co_3_O_4_ ^10^ are often used for high storage supercapacitors. Owing to the large surface area, chemical stability and high conductivity graphene is used as a substrate for the metals and metal oxides.^11,12^ In transition metal oxides, hydroxides and layered materials restrict their applications in supercapacitors, as they produce pseudocapacitance in a lower potential window (*V*).^13–15^ Hence it is crucial to improve the capacitance and energy density to favour the demands of energy storage.

Owing to the dual EDLC and pseudo capacitive nature, high conductivity associating metal nanoparticles and carbon electrodes were employed.^16,17^ Multilayer graphene is used as an electrode to give excellent conductivity in supercapacitors.^18–20^ It has been shown that charging and discharging occurred on the surface of the electrode materials, rather than in the bulk^21^. Owing to the establishment of defect sites, the conduction of the electrons decreases owing to uninterrupted charging and discharging. The decrease in resistance raises the conductivity of the electron between the electrode materials and the current collectors and minimizes the *IR* drop.^22^

Introduction of a heteroatom into the graphene alters the d-band and enhances the electron donation to the parent metals.^23^ Doping of boron onto a sp^2^ bonded hexagonal carbon framework induces a p-type semiconductor, as the boron atom has three valence electrons, one electron less than the carbon atom, which enhances the performance of electrochemical reactions.^4,24^ The electronic transport properties of graphene were improved by incorporation of boron onto the carbon lattice of graphene.^25^ The amount of boron atoms doped into the graphene nanosheets plays a major role in tailoring the band gap energy. Boron doped onto carbon and graphite can improve the coulombic efficiency and energy storage capacitance of anodic materials^26,27^. Long-term cyclic stability for electrochemical retention can be obtained owing to the oxygen containing functional groups in the boron doped graphene nanosheets.^28–31^ Hence, boron doped porous graphene was used to enhance the electrochemical performance of a prepared material that had both pseudo and electric double layer capacitance (EDLC).

The operating voltage of the supercapacitor can be improved to 0–2.0 V by the development of an asymmetric supercapacitor.^32^ An asymmetric solid-state supercapacitor (ASSC) is the combination of the positive material of a battery type pseudo capacitor and the capacitive type negative materials of a porous carbon in the same electrolyte to utilise their different negative and positive electrode potential windows.^33–35^ Among the transition metal oxides CuO is one of the least expensive, has an environmentally friendly nature and has greater stability which can be used to tailor the supercapacitor, giving the electrode materials more intense energy and power densities.^36,37^ CuO used in supercapacitor applications has various nanostructures such as nanorods, nanowires, nanosheets, nanoribbons and flowers.^38–40^

The resistance of copper to oxidation is very poor, hence carbon shells on graphene nanosheets were used to encapsulated the Cu nanoparticles. However, bare nanoparticles of Cu oxidized to Cu_2_O within a few hours at room temperature. The copper nanocore was shielded by the C shells, and protected them from further oxidation. Usually, the conductivity of carbon shells is very poor, hence by using boron the conductivity of carbon is significantly increased and the material has greater stability. Accordingly, single layer C atoms on the Cu passivates the oxidation by an adsorption-diffusion mechanism.^41^ Furthermore, above 1000 °C the Cu nanoparticles melt, hence anchoring of copper on the carbon lattice of graphene is difficult.^42,43^ The transformation of Cu(ii) to the Cu(i) species in the charge/discharge process provides evidence of a redox reaction in the high-performance supercapacitor.

The asymmetric supercapacitor of CuO with activated carbon shows the highest capacitance of 83 Fg^−1^ with the potential window of 1.6 V in KOH.^44^ CuO/CNT and CuO/rGO with a specific capacitance of 137.6 Fg^−1^ and 163.7 Fg^−1^ in a KOH electrolyte was reported by Liu *et al.*^45,46^ and Zhao *et al.* described CuO on graphene with a specific capacitance of 331.9 Fg^−1^ in 6 M KOH as the electrolyte.^47^

To the best of our knowledge, the investigation of copper nanoparticles anchored boron doped graphene nanosheets (CuBG) has not previously been reported, based on our literature review. In the present study, CuBG samples were successfully synthesized using a two-step hydrothermal method followed by pyrolysis of the material in a chemical vapor deposition (CVD) reactor at different temperatures in an Ar atmosphere. Incorporation of boron (heteroatom) on the copper functionalized graphene can be used to produce energy storage devices with a long cyclic stability. Here, we report for the first time the synthesis and characterization of CuBG samples with an excellent electrochemical performance both in H_2_SO_4_/PVA and KOH/PVA solid electrolytes. ASSC devices were fabricated and attained a maximum energy density (132.5 W h kg^−1^) and power density (1000 W kg^−1^) using a H_2_SO_4_/PVA solid electrolyte. Using a KOH/PVA solid electrolyte, the realized energy density and power density was 31.1 W h kg^−1^ and 800 W kg^−1^ respectively.

Herein, we designed and fabricated a symmetric and an asymmetric supercapacitor with copper anchored boron-doped graphene nanosheet as the electrode materials on the graphene sheet with H_2_SO_4_/PVA as the solid electrolyte. This paper highlights the performance of the supercapacitor, which has a specific capacitance of 483 Fg^−1^ at 1 Ag^−1^ in the symmetric supercapacitor and 238 Fg^−1^ at 1 Ag^−1^ in the ASSC with an extended potential window of 2.0 V, these results show that the copper anchored boron doped graphene nanosheet is a promising electrode material for use in an electrochemical supercapacitor. Furthermore, in a single asymmetric device we used a 2 V green color LED to determine the long-life stability, which emits a constant glow of light in the range 0 to 134 s with regular decreases in power. After 140 s an even power was maintained for a small interval of time (in seconds). This shows the high energy and power density can transfer electrons much faster between the electrode materials and within the solid electrolytes of the flexible ASSC device.

## Materials and methods

### Materials

Graphite powder (∼25 μm), concentrated sulphuric acid (H_2_SO_4_), potassium permanganate (KMnO_4_), sodium nitrate (NaNO_3_), concentrated hydrochloric acid (HCl), boron trioxide (B_2_O_3_) and copper sulphate pentahydrate (CuSO_4_·5H_2_O), were purchased from Sigma Aldrich-India for the synthesis of GO and CuBG, all were used without further purification. 2D water was used for the entire preparation and purification process.

### Synthesis of graphene oxide

Graphene oxide was prepared by using synthetic graphite powder as a starting material (<20 μm, with a purity of 99.99 wt%, Aldrich). The GO synthesis process is described as follows: 1 g graphite powder, 0.5 g sodium nitrate (NaNO_3_) and 23 ml of concentrated sulfuric acid (H_2_SO_4_) were added into a 500 ml round bottom flask kept at 5 °C in an ice bath with constant stirring for 15 min. Then, 3 g of potassium permanganate (KMnO_4_) was added slowly into the flask. The reaction temperature was maintained for 2 h at 5 °C and then the reaction temperature was slowly raised to 35 °C and maintained for another 30 min with vigorous stirring. Deionized (DI) water (46 ml) was added to the suspension and the temperature was increased to 98 °C. The mixture was kept at the same temperature with continuous stirring for 30 min. To complete the reaction 140 ml of DI water and 10 ml of hydrogen peroxide (10% v/v) were added. A yellowish-brown product was obtained. The resulting product of GO was washed with 5% diluted HCl solution. To remove the acid and Mn ions, the solution was warmed at 70 °C respectively. The product was later centrifuged and dried at 60 °C for 24 hours.

### Synthesis of copper anchored boron doped graphene nanosheet

Copper sulphate (10 mg) was dissolved in 100 ml of ethanol with mechanical stirring for two days and 10 mg of B_2_O_3_ was added to the solution and allowed to stir for 2 h to completely dissolve the particles, 10 mg GO was added to the solution and it was sonicated for 30 min to achieve the complete dispersion of all particles. The mixtures were transferred into an autoclave and heated at 150 °C for 12 h. To remove the unreacted and amorphous substances, the product was washed with DI water several times and dried at 60 °C for 12 h. CuBG1 was prepared by heating the obtained product at 150–550 °C in a CVD reactor under an Ar atmosphere. The product obtained was washed using DI water and dried at 60 °C for 12 h. Similarly, CuBG2, CuBG3, CuBG4 and CuBG5 were prepared at the different temperatures of 650 °C, 750 °C, 850 °C and 950 °C respectively. Above 1000 °C the material becomes crystalline.

### Characterization techniques

#### Material characterization

The as synthesized materials were analysed using X-ray diffraction (XRD) on a PANalytical High Resolution XRD (PW 3064/60) in a 2*θ* range from 5 to 90° with Cu Kα radiation (*λ* = 1.5406 Å) operated at 30 mA, 40 kV to give structural conformation. Raman spectra were conducted using a confocal Raman microscope (Model WITech GmbH, CRM Alpha 300S) at room temperature and a charge coupled device (CCD) detector with a helium neon laser wavelength of 633 nm. The microstructure and the morphology of graphene oxide and Cu–B-doped GO were canvased using a field emission scanning electron microscope (FESEM) FEI Quanta FEG 200 at an accelerating voltage of 3.0 kV and high resolution transmission electron microscopy (HRTEM, S-Twin F20 TECHNAI G^2^). The elemental composition on the surface and the chemical nature of the samples were studied using X-ray photo electron spectroscopy (XPS) using a Kratos analytical spectrometer with a monochromatic Al Kα radiation of 10 mA and 15 kV. According to the C 1s peak at 284.6 eV all of the spectra were corrected. The background subtraction and curve fitting were carried out using Casa XPS software.

#### Electrochemical measurements

The solid-state symmetric and asymmetric supercapacitor electrochemical properties, such as cyclic voltammetry (CV), galvanostatic charge–discharge (GCD) and electrochemical impedance spectroscopy (EIS)-Nyquist plot of the as-synthesized copper nanoparticles anchored on the boron doped graphene nanosheet were investigated using an Autolab-PGSTAT302N electrochemical work station with H_2_SO_4_/PVA as a solid electrolyte. The electrode active materials were prepared by making a paste of CuBG and polyvinylidene fluoride (PVDF) in a mass ratio of 9 : 1 in the absence of any other conducting material, 1 ml of *N*-methyl-2-pyrrolidone (NMP) was added and the material was ground well in a mortar and pestle for 10 min to attain a homogeneous slurry. The prepared activated slurry was painted onto the graphite sheet evenly and allowed to dry at 80 °C overnight. Using a similar method the symmetric supercapacitor was prepared by sandwiching CuBG4 and CuBG5 with H_2_SO_4_/PVA as the solid electrolyte, additional care must be taken while preparing the symmetric device. From these, the capacitance was calculated in the two electrode system, and the mass ratio of the electrode was obtained using the charge-balance theory.

Cyclic voltammetry was performed in the potential window of −0.4 to 0.2 V at a different scan rate. The gravimetric capacitance of the two electrode system was calculated using the formula:1
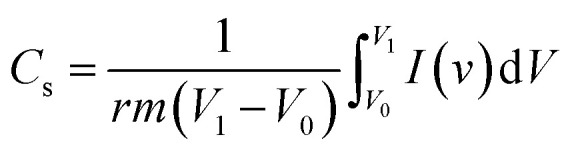
In which, *r* is the scan rate (mV s^−1^), *m* is the mass of active material (g) in the electrode, *V*_0_, *V*_1_ are the initial and final voltage windows (V) and *I* is the current (A).

A charge–discharge experiment was conducted in different potential windows at different current densities of 1, 2, 3, 4, 5, 10 and 20 Ag^−1^. The specific gravimetric capacitance (*C*_s_) was calculated using eqn (2)^48^2
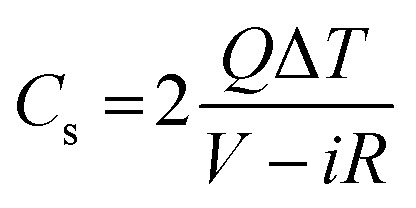
In which, *Q* is the current density during the discharge process (A), Δ*T* is the discharge time (s), *V* is the potential window difference and *iR* is the internal resistance voltage drop at the beginning of the discharge.

The energy density (*E*, W h kg^−1^) and power density (*P*, W kg^−1^) were calculated using the specific capacitance obtained from the charge–discharge.3
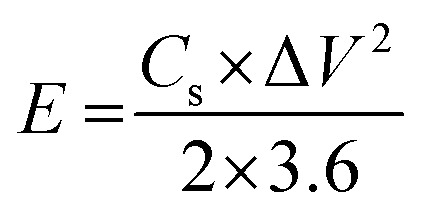
4
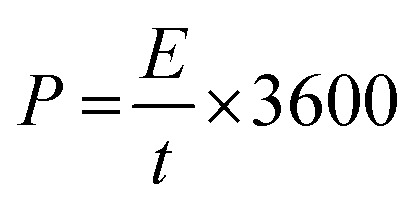
In which, *t* is the discharge time.

#### Synthesis of H_2_SO_4_/PVA and KOH/PVA electrolyte

The solid electrolyte of H_2_SO_4_/PVA used for the device was prepared by adding 3 ml of H_2_SO_4_ into 30 ml of distilled water under constant stirring, 3 g of polyvinyl alcohol powder (PVA) was then added to the solution. The mixture was stirred at 85 °C until a clear solution was obtained and then allowed to cool to room temperature in a Petri dish. Similarly, 4 g of KOH and 6 g of PVA powder was added to 20 ml of distilled water at 85 °C to give formation of a gel.

#### Fabrication of symmetric and asymmetric supercapacitors

The flexible solid-state asymmetric supercapacitor was assembled with graphene nanoplatelets as the positive electrode and copper nanoparticles anchored on a boron doped graphene nanosheet as the negative electrode. The working electrode was prepared by using H_2_SO_4_/PVA as the solid-state electrolyte and separator. From the preliminary investigation of the prepared samples, CuBG5, CuBG4 and CuBG3 were used to fabricated the ASSC. Initially the surface of the graphite sheet was activated by treating the sheet under ultrasonication for 15 min in ethanol and it was dried at 60 °C for 15 min. The slurry was prepared by mixing CuBG and PVDF in the mass ratio of 9 : 1, and was ground well to achieve a uniform mixing before 1 ml of NMP was added and it was mixed for a further 10 min. The paste of the slurry was coated onto the sheet evenly and dried at 80 °C for 1 h. Similarly, the graphene nanoplatelets were also coated on another sheet. Both the ionic electrodes were sandwiched with a H_2_SO_4_/PVA electrolyte under 1 atm pressure. The thickness between the two electrodes was 1 mm, allowing the device to obtain an interface between electrodes for a day. For the design of the symmetric supercapacitor device, copper nanoparticles anchored onto a boron doped graphene nanosheet were employed as both the positive and negative electrode and the fabrication procedure was same as that described above.

## Results and discussions

### Positive electrode material

**Fig. 1:**
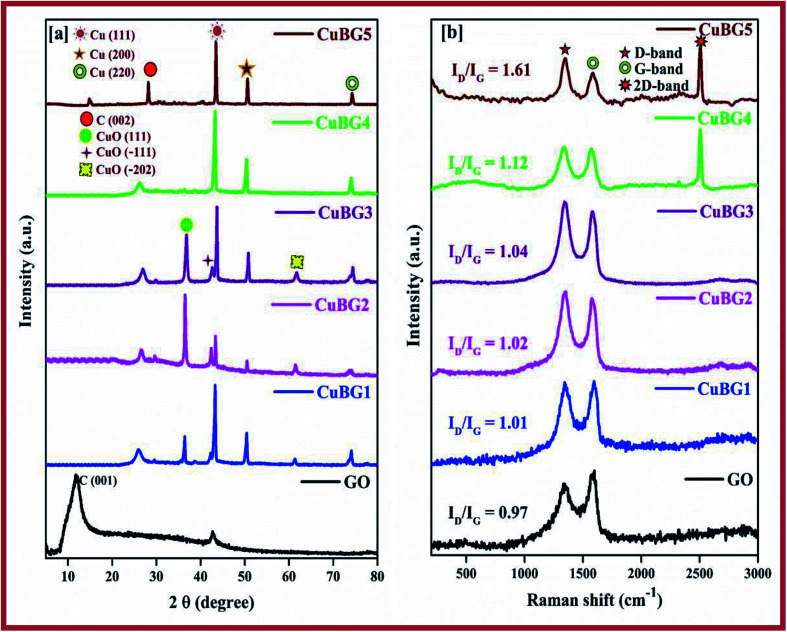
(a) Wide angle XRD spectra of GO, CuBG1, CuBG2, CuBG3, CuBG4 and CuBG5. (b) Raman spectra of GO, CuBG1, CuBG2, CuBG3, CuBG4 and CuBG5.

The XRD pattern for GO gives a peak at 2*θ* = 11° corresponding to a (001) reflection (Fig. 1a). The *d*-space of GO was calculated to be 0.79 nm, which is much higher than graphite, this is due to the harsh oxidation of the graphite. The insertion of an oxygenated functional group on the graphite is attributed to the interlayer space of the GO.^49^ The structure of the crystal and the crystalline size of the prepared nanoparticles were investigated using the wide angle XRD pattern shown in Fig. 1a. From the spectra, the clear and strong peak for CuBG4, CuBG5 at 2*θ* = 43.5°, 50.6°, 74.27° corresponds to (111), (200) and (220) (JCPDS-04-0836), which indicates the formation of highly pure cubic crystalline Cu^50^ nanoparticles on the graphene nanoplatelets. The XRD pattern for CuBG1, CuBG2 and CuBG3 not only shows the high crystalline peak (111), but also (200) and (220) of the cubic crystalline Cu and also the minor diffraction peaks at 2*θ* = 35.5°, 38.8° and 61.4° correspond to the formation of a monoclinic phase of CuO (JCPDS: 05-0661).^37,51,52^

The XRD pattern confirms the formation of highly crystalline CuO for the samples prepared at 550 °C, 650 °C and 750 °C from their corresponding peak positions. The samples prepared at 850 °C and 950 °C reveal metallic Cu in their structure. There is no other impurity peaks and secondary phases were obtained for the prepared CuBG samples. From the XRD pattern, it is noted that the sample that was prepared above 850 °C shows only metallic copper and the peak at 24° also confirms the presence of boron doped graphene. At a high temperature (>850 °C) all of the copper based oxide materials become reduced even in an air atmosphere, owing to the carbon shell encapsulation of the Cu nano metal particles. Here, the copper nanocore was shielded by the C shells and this protected them from further oxidation. Accordingly, the single layer C atoms on the Cu passivates the oxidation at high temperatures by an adsorption-diffusion mechanism.^41^ A further increase in temperature, that is, above 1000 °C, and the Cu nanoparticles melt, hence the anchoring of copper on the carbon lattice of graphene is difficult.^42,43^ Using the software XRDA 3.1 the lattice parameters were estimated for the corresponding peaks which were found to be *a* = *b* = *c* = 3.5925 ± 0.0075 Å with cell volume *V* = 46.367 Å^3^ for CuBG5, 3.6219 ± 0.0020 Å with cell volume *V* = 47.512 Å^3^ for CuBG4, 3.5943 ± 0.0075 Å with cell volume *V* = 46.436 Å^3^ for CuBG3, 3.6205 ± 0.0140 Å with cell volume *V* = 47.459 Å^3^ for CuBG2 and 3.6191 ± 0.0015 Å with cell volume *V* = 47.403 Å^3^ for CuBG4. Using the Debye–Scherrer equation the crystallite sizes of the prepared samples were calculated using the formula:

5 *D* = *kλ*/*β* cos *θ*

In which, *D* the average crystallite size, *β* the full width at half maximum of the individual peak, *k* is the shape factor and *θ* the diffraction angle. The approximate crystallite size calculated using the Debye–Scherrer equation is 24 nm, which was further confirmed using HRTEM particle size analysis.

HRTEM particle size analysis was used to distinguish the disordered and ordered structure of the C–C bonds, carbon–metal bonds and carbon heteroatom. It is evident from Fig. 1b, that two peaks were obtained at 1334.58 cm^−1^ and 1559.4 cm^−1^, indicating the incorporation of the metal and boron on the graphene sheet. The defects in the carbon materials were reflected in the D/G ratio. The *I*_D_/*I*_G_ ratio of the CuBG1, CuBG2, CuBG3, CuBG4 and CuBG5 are 1.0, 1.01, 1.033, 1.21 and 1.6 cm^−1^ respectively, which was evidently larger than graphene oxide at 1.0 cm^−1^ respectively, this ratio demonstrated that metal and boron atom insertion causes structural defects on the graphene sheet. The small peak at 2504.35 cm^−1^ indicates the multilayer of the graphene sheet, in comparison, CuBG4 and CuBG5 show a large 2D band which reflects the formation of the multi-layer compounds. This was further confirmed from the FESEM and HRTEM analysis.

**Fig. 2:**
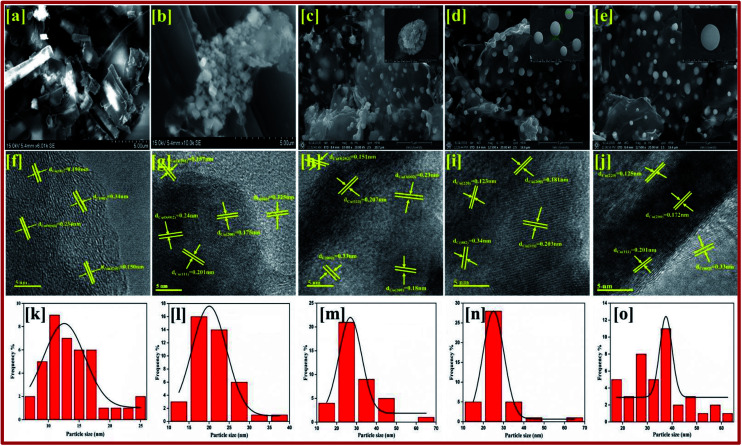
FESEM images of (a) CuBG1; (b) CuBG2; (c) CuBG3; (d) CuBG4; and (e) CuBG5. HRTEM images of (f) CuBG1; (g) CuBG2; (h) CuBG3; (i) CuBG4; and (j) CuBG5 at high magnification. (k–o) Particle size distribution histogram of prepared CuBG samples.

The qualified morphologies of CuBG1 obtained using FESEM are shown in Fig. 2a. This shows the presence of a large number of copper nanoparticles on the graphene nanoplatelets. The small nanorods of the copper and boron particles have clearly migrated all over the sheets. In Fig. 2b, CuBG2 appears with a large number of carbon nanoparticles in the shape of rods in the graphene sheet and boron balls become attached inside the nanoplatelets of graphene. Fig. 2c–e shows the uniform morphology of the dandelion and global shaped copper nanoparticles on the graphene nanoplatelets. The dandelion shaped single particles are shown in the inset image. From CuBG3, CuBG4 and CuBG5, as the temperature increases the insertion of copper and boron nanoparticles increases with regular separation of the particles without the occurrence of aggregation on the graphene nanoplatelets.

From Fig. 2f–j it can be seen that the copper particles migrated onto the graphene sheet regularly, whereas the large particles were framed on the sheet owing to the separation of the individual particles during calcination at high temperatures. The fringes of the metal particles on the sheet can be clearly observed in the high-resolution images. From the measured particle size, it can be seen that most of the particles are in the range of 20–30 nm and also a large number of smaller particles are dispersed on the sheet. In the higher magnification, patches of the metals and fringes of boron on the graphene sheet were identified. From the TEM images at a low magnification range of 50 nm, the particles size was calculated using ImageJ software and cumulative distribution of the particle size on the graphene sheet is given in the graph shown in Fig. 2k–o. It can be seen from Fig. 2n, that CuBG4 has a regular distribution of particles at a particle size of 24 nm. Using the high magnification images the interlayer distance between the fringes was also calculated to be 5 nm using ImageJ software and this correlated with the XRD *d*-spacing. In addition, using the selected areas electron diffraction (SAED) pattern the *hkl* planes were identified from their corresponding *d*-spacing calculation using the relationship:

6 *d*_*hkl*_ = *Lλ*/*r*

in which, *d* represents the inter-planar *d*-spacing, *L* represents the distance between the camera and the sample, *λ* is the wavelength of the incident electron beam used in the analysis and *r* is radius of the diffraction rings in the SAED pattern.

**Fig. 3:**
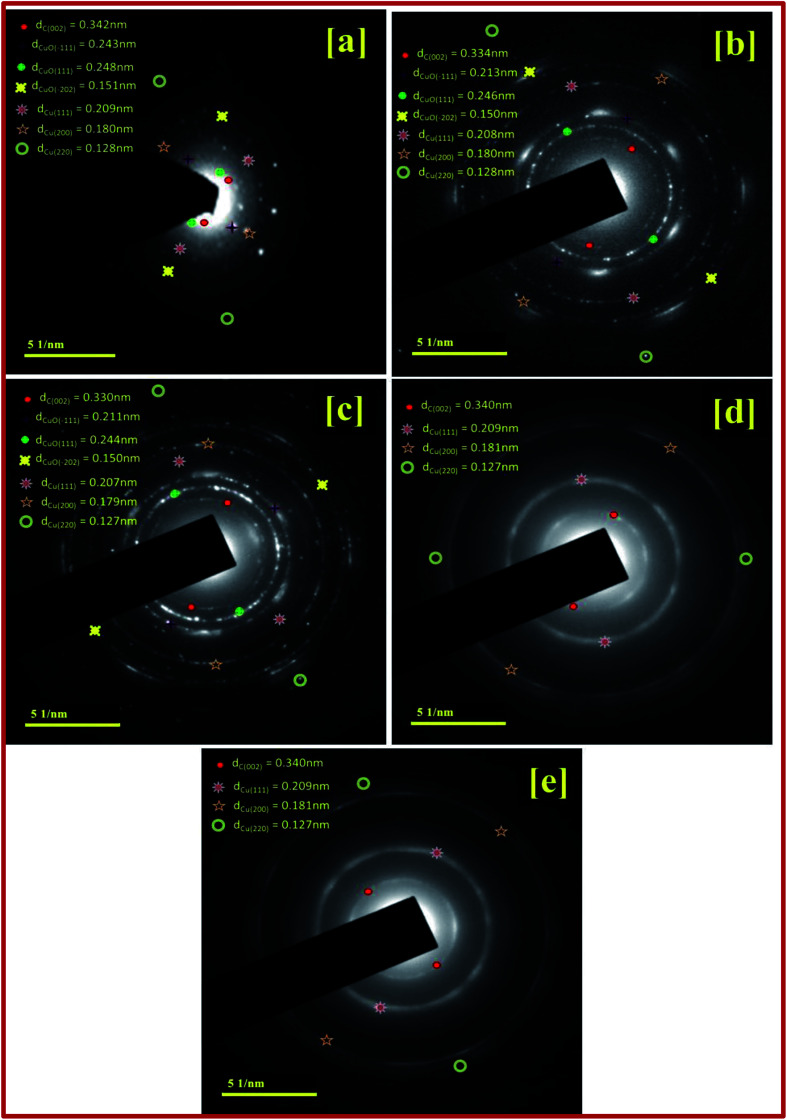
SAED pattern for: (a) CuBG1; (b) CuBG2; (c) CuBG3; (d) CuBG4; and (e) CuBG5.

The SAED pattern in the Fig. 3 shows the high crystalline nature with different rings and clearly indicates the transformation of the particles. CuBG4 and CuBG5 show only three circles corresponding to the carbon (002), copper (111) and copper (200) plane. Whereas, the other samples of CuBG1, CuBG2 and CuBG3 show different circles, which correspond to the carbon (002), Cu (111), Cu (200), CuO (111) and CuO (−111). Thus, the transformation of materials at different temperatures was further confirmed using the HRTEM images.

**Fig. 4:**
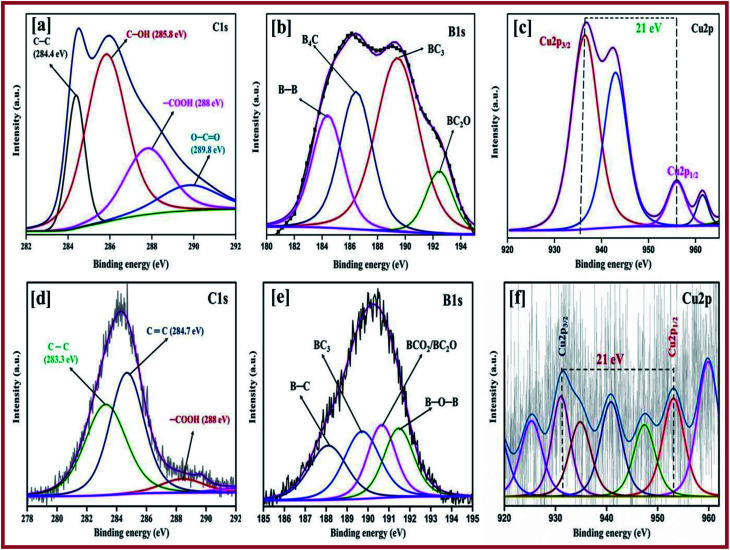
Deconvoluted XPS spectra of C 1s of (a) CuBG3 and (d) CuBG4; B 1s of (b) CuBG3 and (e) CuBG4; and Cu 2p of (c) CuBG3 and (f) CuBG4.

The chemical composition and valence state of the elements were evaluated using the patterns obtained using X-ray photoelectron spectroscopy (XPS), which were similar for the copper and boron graphene nanoplatelet catalysts with two different temperature of 750 °C and 850 °C. The high resolution deconvoluted Cu 2p spectrum is predicted in Fig. 4c and f. The high intense peak at a binding energy of around 953.0 eV represents Cu 2p_1/2_ and the peak for Cu 2p_3/2_ appearing at a lower energy, 932.5 eV, respectively, can be attributed to the Cu^+^/Cu^0^ ^8,53^ state, which confirms that the copper exists on the sheet of graphene as the metallic phase. In contrast, the peaks at 933.4 eV and 953.8 eV corresponds to the Cu^2+^ characteristic peaks at Cu 2p_3/2_ and Cu 2p_1/2_ respectively, which shows that CuO is present on the graphene sheet.^54^ Further peaks at the higher binding energies of 941.6 eV and 961.8 eV imply the presence of Cu^2+^ ions,^55^ which indicates the presence of CuO, whereas the Cu^2+^ of cupric oxide in the 3d^9^ configuration appears as the hole in the 3d band. The Cu^+^ of cuprous oxide has a completely filled d-orbital (3d^10^), only the 4 s band is unoccupied.^56^ The fine gap between the Cu 2p_1/2_ and Cu 2p_3/2_ is about 21 eV, which provides evidence for the CuO spectrum.^57^ This determination also corresponds to the X-ray diffraction patterns.

Fig. 4a and d show the C 1s peak, and Fig. 4b and e show the B1s peak of CuBG3 and CuBG4. Using the Gaussian Lorentz peak, curve fitting was carried out after correction of the Shirley-background. The peak at a binding energy of 284.3 eV was assigned to C=C. The chemical shift of 0.8–1.3 eV was assigned to the hydroxyl group of C–OH, the peak shift at 4–5 eV corresponds to the acid (–COOH) group, the carbonyl group (C=O) was assigned to the shift of 2.65–3.45 eV and the shift of peak from 0.0–5.5 eV was assigned to the carbonate group (O=C=O) group, respectively.^25,58,59^ The peak ratio of C=C in the graphene sheets decreases with an increase in the conjugation of the oxygen functional group with the boron and copper nanoparticles. Owing to the harsh oxidation on graphite, the functional group of the oxygen becomes incorporated on C–C, which has already been proved using XRD.

The peak at 187.2 eV is assigned to B–C and the peak at 188.9 eV corresponds to B_4_C and the boron substituted carbon.^60,61^ The presence of the B 1s peak at 186 eV confirms the intercalation of boron on the graphene sheet. The B 1s peak of CuBG3 has the highest peak at 186.4–189.36 eV and this is deconvoluted into four small peaks at 185.6, 186.4, 189.4 and 192 eV which corresponds to the clusters of boron (185.3 eV), B_4_C (186.4 eV) and BC_3_ (189.4 eV) respectively.^62,63^ The single peak at a higher binding energy of 192.6 eV corresponds to the oxides of boron BC_2_O and BCO_2_ respectively.^64^ The B1s peak of CuBG4 shows the highest peak at 190.32 eV and four small peaks at a high binding energy of 189.7, 188.1, 190.6 and 191.4 eV respectively, which confirms the replacement of the carbon atom in the honey comb lattice by boron (188.1 eV), BC_3_ (189.7 eV) and the oxides of boron BCO_2_/BC_2_O (190.6 eV), respectively. Thus, the XPS confirms the incorporation of the boron atom on the carbon lattice of the graphene sheets, the peaks at a higher binding energy prove that the boron is bonded with the carbon and oxygen atoms.^65^ The deconvoluted XPS peak of oxygen is shown in Fig. S1.†

### Electrochemical performance of the CuBG electrodes in H_2_SO_4_/PVA

#### Negative electrode materials

The electrochemical cyclic performance and capacitance of the electrode material CuBG were evaluated using CV and GCD techniques in a two-electrode system with H_2_SO_4_/PVA as the solid electrolyte at different electric current densities (1 Ag^−1^ to 20 Ag^−1^) with a potential difference of −0.4 to 0.2 V.

It can be seen from Fig. 4a that the redox peaks on the CV impose the performance of double-layered capacitance. The CV curve of the prepared samples are shown in Fig. S2.† The shifting of the anodic peak from 0.012 to 0.08 V corresponds to the copper oxidation from Cu(i) to Cu(ii) as the scan rate decreases from 100 to 10 mV s^−1^ and cathodic peaks shifts from −0.06 to −0.012 V and imparts the reduction of Cu(ii) to Cu(i) respectively. The CV curve of the sample shows the ideal rectangular curve of EDLC, whereas boron on the graphene sheet produces a pseudocapacitance behaviour. From Fig. S2,† as the scan rate increases there is no significant change in the shape of the CV curve, which indicates the excellent conductivity of the electrode materials.^40^ As the scan rate increases, the cathodic peak shifted towards a more negative direction, this is the internal resistance of the electroactive material. The different charge storage mechanisms that take place on the surface of the electrode are based on the adsorption/desorption of H^+^ ions on the surface.Cu → Cu^2+^ + 2e^−^Cu^2+^ + e^−^ → Cu^+^2CuO + 2H^+^ + 2e^−^ → Cu_2_O + H_2_O

For the materials prepared at a high temperature of 850 °C and 950 °C, metallic copper also plays a major role in the CV and charge–discharge curve. At a low temperature of 550, 650 and 750 °C, the boron and oxygen containing groups in boron and copper play a vital role, and hence the materials prepared at low temperature show a EDLC layer. At high temperature, the copper oxide and copper hydroxide gets transformed to metallic Cu and hence both EDLC and pseudo capacitance exists in CV. The anodic and cathodic peak of CuBG4 was larger than that of CuBG5, which indicates the oxidation of Cu to Cu(ii) and the reduction of Cu(ii) to Cu(i). The peak at the potential of 0.1 V indicates oxidation of metallic Cu to Cu^2+^.

The resistivity of copper is 1.68 × 10^−8^ Ωm, whereas the conductivity of copper is 5.96 × 10^7^*σ* (S m^−1^) which has a much higher conductivity than the semiconductor CuO. The highly pure copper gives 100% conductivity, and metallic copper is more stable than unstable CuO and very unstable Cu_2_O.

**Fig. 5:**
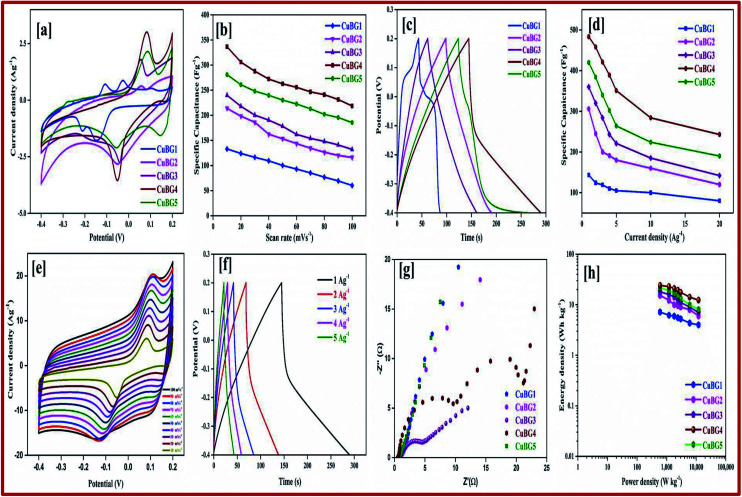
(a) CV of all CuBG samples at a scan rate of 2 mV s^−1^. (b) Variation of specific capacitance of the CuBG samples at different scan rates. (c) Charge/discharge curve of CuBG samples at 1 Ag^−1^. (d) Specific capacitance of samples at different current densities from 1 Ag^−1^ to 20 Ag^−1^. (e) CV of CuBG4 at different scan rates of 100–10 mV s^−1^. (f) Charge/discharge curve of CuBG4 at different current densities from 1 to 20 Ag^−1^. (g) EIS-Nyquist plot of all CuBG samples. (h) Energy and power density of all samples calculated from the discharge time at different current densities.

Evidently, in Fig. 5a the anodic and cathodic peak of CuBG4 was larger than the other, which indicates the oxidation Cu to Cu(ii) and the reduction of Cu(ii) to Cu(i). The conductivity of metallic copper is higher compared to the semiconductors of CuO, Cu_2_O and Cu(OH)_2_.

By calculating the integral area from the curve, the specific capacitance was calculated and plotted against the different scan rates in Fig. 5b. From the figure it can be seen that as the scan rate increases there is a constant decrease in the specific capacitance. Comparing the area of the electrode materials, CuBG4 shows a larger area than the others. At the scan rate of 100 to 10 mV s^−1^ the specific capacitance of CuBG5 increases from 186 to 281 Fg^−1^, the capacitance of CuBG4 increases from 219 to 336.6 Fg^−1^, CuBG3 from 132 to 240 Fg^−1^, CuBG2 from 116 to 214 Fg^−1^ and CuBG1 from 60 to 133 Fg^−1^, respectively. This shows that as the scan rate decreases the specific capacitance increases, which is assigned to the diffusion effect of the electrolyte ions.^66^

Fig. 5c presents the galvanostatic charge/discharge curve of the CuBG at different temperatures at the current density of 1 Ag^−1^, the charge/discharge curves of all of the samples are shown in Fig S2† at current densities from 1 to 10 Ag^−1^ respectively. From the charging curve, the high capacitance yield in the −0.4 to 0.2 V potential window corresponds to the oxidation of Cu(i) to Cu(ii) as evidenced by the CV curve. In the discharge curve there are two regions with a fast *IR* drop potential and a slow decay potential.

The pseudocapacitive nature of Cu in the CuBG electrodes is evidenced in the charge/discharge of the non-linear curve. The longer charge/discharge of the CuBG4 indicates a higher specific capacitance of 483 Fg^−1^ compared to CuBG5 (420 Fg^−1^), CuBG3 (360 Fg^−1^), CuBG2 (306 Fg^−1^) and CuBG1 (143 Fg^−1^) at 1 Ag^−1^ respectively. The specific capacitances were calculated for the samples at different current densities and are plotted in Fig. 5d. From the graph it can be seen that as the current density increases there is a gradual decrease in the specific capacitance owing to the increase in the *IR* drop and at high current densities, demonstrating that the active materials were insufficient in the redox reaction^67^. The cyclic stability of the electrode materials was evaluated using GCD at 5 Ag^−1^ for 5000 cycles. Fig. S3† shows the capacitance retention and coulombic efficiency of CuBG1 (a), CuBG2 (b), CuBG3 (c), CuBG4 (d) and CuBG5 (e) at different cycle numbers. The capacitance of CuBG4 decreases from 351 to 331 Fg^−1^ after 5000 cycles. The specific capacitance decreases gradually, this is due to the interaction of the copper nanoparticles and boron on the matrix of the graphene sheet. The application of electrode materials for supercapacitor devices is calculated using the energy density and power density.

The kinetics of the interfacial charge transport mechanism between the electrodes was analyzed in the two-electrode system in the frequency range of 1 MHz to 0.1 Hz for an applied open circuit potential (OCP) at room temperature. Fig. 5g represents the EIS-Nyquist plots of the prepared samples (CuBG1, CuBG2, CuBG3, CuBG4 and CuBG5) which shows two semicircle trends for the corresponding two interfacial charge transport processes. The first semicircle in the high frequency region is attributed to total charge transport resistance (*R*_ct1_) at the electrolyte/electrode material interface and the mid frequency semicircle contributes to the total charge transport resistance (*R*_ct2_) at the interface of the CuBG grain interior. The intersection of the semicircle on the real part of the impedance provides the distinct interfacial charge transport resistance of the samples. *R*_s_ represents the combined effect of the surface resistance of the electrode (carbon felt) and the diffusion resistance of the electrolyte ions, *C*_μ_ is the total chemical capacitance and *W*_s_ is the Warburg diffusion resistance. Here, the obtained semicircles were fitted using equivalent circuit modelling and the parameters of the electrochemical fit are summarized in Table 1.

**Table 1:** Electrochemical parameters for the EIS-Nyquist plot

Sample	*R*_s_ (Ω)	*R*_ct1_ (Ω)	*R*_ct2_ (Ω)	*C*_μ_ (F)	*W*_s_	χ^2^
CuBG1	0.94	13.79	42.83	0.037	0.152	5.96 × 10^−3^
CuBG2	0.93	1.99	13.86	0.174	0.121	5.43 × 10^−3^
CuBG3	0.91	5.54	9.53	0.324	1.137	5.80 × 10^−3^
CuBG4	0.69	0.43	32.61	0.101	0.126	2.30 × 10^−3^
CuBG5	0.85	11.73	12.63	0.073	0.157	1.63 × 10^−3^

It is noted from Table 1, that *R*_s_ appears to be equal owing to the utilization of the same carbon felt as the electrode material for all of the prepared devices. Furthermore, the charge transport resistance (*R*_ct2_) at the electrolyte/electrode material interface is very low in CuBG4 compared to the other samples which reveals efficient charge transportation in the CuBG4 material/electrolyte interface. This is responsible for the high specific capacitance obtained by the EDLC behavior in the CuBG4 based asymmetric supercapacitor device. However, *R*_ct2_ seems to be very high for all of the samples (CuBG) owing to the charge transport resistance at the grain interior. Although the *R*_ct2_ for the CuBG4 sample is high, its specific capacitance is greater than the other samples owing to the presence of low *R*_ct1_. In addition to the pseudo-capacitive nature of the CuBG4 material, the low *R*_ct1_ offers a high EDLC behaviour which is responsible for the high specific capacitance in CuBG4. The change in the charge transport resistance before and after the cycle stability test was evaluated using the EIS-Nyquist plot.

The Ragone plot shows the energy and power density of the materials, this is an important parameter for determining the performance of the supercapacitor devices. Fig. 5h shows the power density *versus* energy density at various current densities for all of the symmetric devices. From the plot it is evident that CuBG4 shows the highest energy density of 24.13 W h kg^−1^ at a power density of 600 W kg^−1^ at 1 Ag^−1^, which is higher than the other devices of CuBG5 21.6 W h kg^−1^, CuBG3 18 W h kg^−1^, CuBG2 15.31 W h kg^−1^ and CuBG1 7.1 W h kg^−1^ at the same power density of 600 kW kg^−1^ calculated from the discharge area of the current density 1 Ag^−1^. Thus, this result establishes that CuBG4 has a high energy and power density in a wide potential window, which is suitable for use in the supercapacitor devices.

#### Positive electrode material

**Fig. 6:**
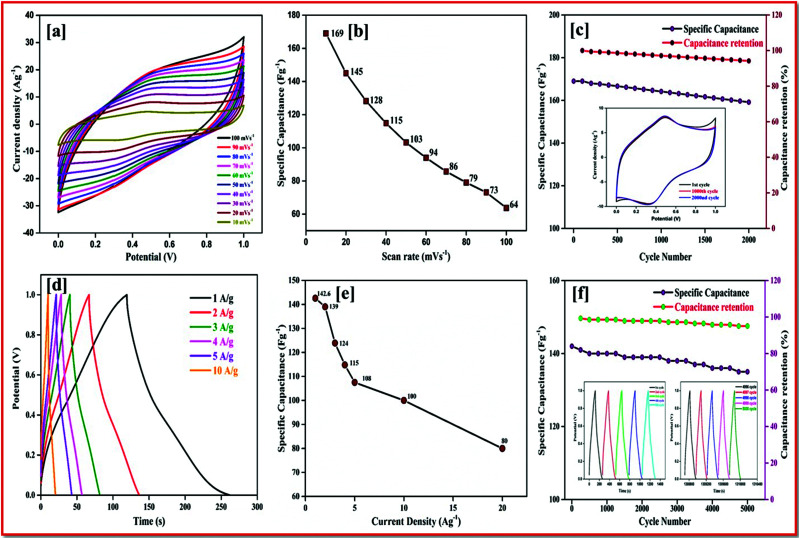
(a) CV of rGO at different scan rates. (b) Specific capacitance at different scan rates from 100 to 10 mV s^−1^. (c) Specific capacitance and capacitance retention calculated using the CV curve, cyclic stability for 2000 cycles. (d) Charge/discharge curve of rGO at different current densities. (e) Specific capacitance at different current densities. (f) Specific capacitance and capacitance retention for a long cyclic stability of 5000 cycles at 5 Ag^−1^.

In the ASSC, rGO have been used as the anode material, as they have a high surface area and are low cost. The performance of the rGO electrode material was tested in three electrode supercapacitors with platinum wire as the counter electrode and Ag/AgCl as the reference electrode. Fig. 6a shows the cyclic voltammetry of rGO at different scan rates from 100 to 10 mV s^−1^ in the potential range of 0–1.0 V. The quasi-rectangular shape of the CV curve indicates the charge stored in the electrode dominated the high double layer capacitance. The capacitance was calculated by integrating the CV curve. The retainment of the rectangular shape even at a lower scan rate without any distortion shows a splendid rate performance implicit in the fast-discharge property of graphene. Reduced graphene nanoplatelets show a capacitance of 169 Fg^−1^ in an electrolyte owing to the correlation of the high energy density with a small pore size and high-power density with a large pore size. Moreover, rGO shows a specific capacitance of 169, 145, 128 and 103 Fg^−1^ at different scan rates of 10, 20, 30 and 50 mV s^−1^ (Fig. 6b) respectively. Fig. 6c shows the cyclic stability of rGO at the 1^st^, 1000^th^ and 2000^th^ cycles, this confirms a capacitance retention of 94% after 2000 cycles.

Fig. 6d shows the GCD of rGO at different current densities of 1, 2, 3, 4, 5 to 10 Ag^−1^. The equilateral triangular charge/discharge of rGO shows the high stability of the materials. Fig. 6e shows a regular decrease in the specific capacitance with an increase in the current density. The cyclic stability has also been calculated at 1 Ag^−1^ for 5000 cycles. The first and last five cycles are plotted inside the capacitance retention graph plotted in Fig. 6f, this produces a 95% retention with a decrease of specific capacitance from 142 Fg^−1^ to 135 Fg^−1^.

#### Asymmetric solid-state supercapacitor in H_2_SO_4_/PVA

Regarding the redox character, the composites of CuBG nanoparticles have a high capacitance compared to rGO owing to the fast-ion transport. Using these two materials, positive and negative electrodes were fabricated for an asymmetric supercapacitor, respectively. In both the two and three electrode materials the electrochemical properties were evaluated with a static window potential for the electrode materials of rGO for the synthesized materials. In the asymmetric supercapacitor, the copper–boron-doped graphene oxide acts as a cathode and the graphene oxide as an anode, which is denoted as rGO//CuBG. By combining the two electrodes of rGO and CuBG, the cyclic voltammetry of each electrode was estimated in a two-electrode system with stable potential windows of 0–1 V for rGO and −0.4 to 0.2 V for CuBG, which meant that the constructed device of rGO//CuBG accomplished a potential window of 2 V. According to the equation, 1/*C*_total_ = 1/*C*_cathode_ + 1/*C*_anode_, the positive and negative electrode charge storage capacitances were balanced to produce a maximum capacitance for the fabricated device. As the positive electrode (rGO), negative electrode (CuBG) potential window and specific capacitance were different, the loading of the mass on these two electrodes was adjusted to balance the charge storage capacity. Using the charge balance theory (*Q*_+_ = *Q*_−_), the negative to positive mass ratio was calculated using the following equation:^68,69^

7 *m*_+_ × *C*_s+_ × Δ*V*_+_ = *m*_−_ × *C*_s−_ × Δ*V*_−_

8 *m*_+_/*m*_−_ = (*C*_−_ × Δ*V*_−_)/(*C*_+_ × Δ*V*_+_)

in which, *Q*_+_, *Q*_−_, *m*_+_, *m*_−_, *C*_+_, *C*_−_, Δ*V*_+_, Δ*V*_−_ are the charges, mass, capacitance and potential windows of the (positive) anode and (negative) cathode obtained in the symmetric two electrode and three electrodes.

**Fig. 7:**
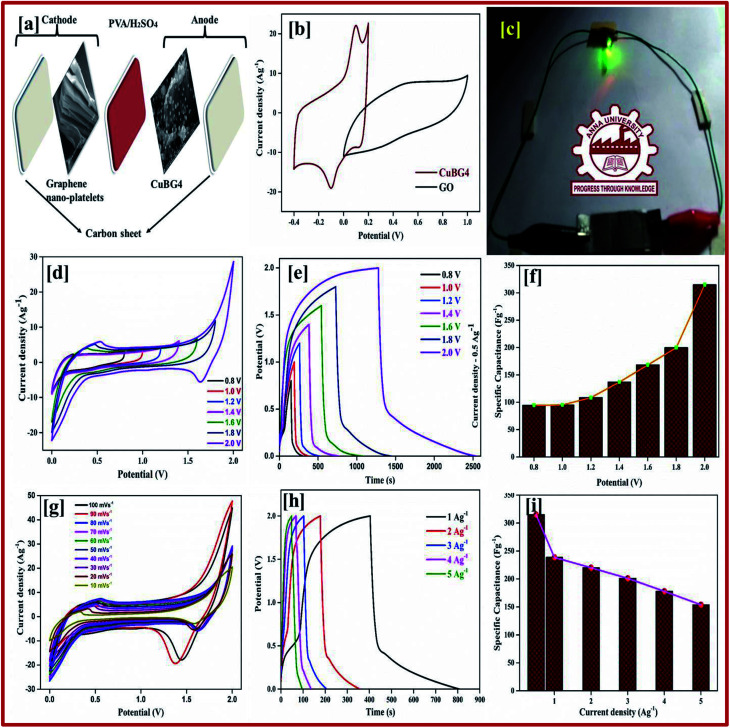
(a) Schematic diagram of CuBG4. (b) CV of rGO//CuBG4 device at 50 mV s^−1^. (c) 2 V LED being illuminated with an ASSC device. (d) CV curve of CuBG4 at different potentials from 0.8 to 2.0 V. (e) Charge–discharge curve of CuBG4 at different potentials from 0.8 to 2.0 V. (f) Specific capacitance of CuBG4 at different potential windows. (g and h) CV and charge–discharge curve of CuBG4 at different scan rates in a high potential of 2.0 V; and (i) the specific capacitance of CuBG4 at different current densities from 1 to 20 Ag^−1^ in the highest potential of 2.0 V.

Fig. 7b shows the CV curve of the fabricated asymmetric solid-state device at different potential voltages at a scan rate of 50 mV s^−1^ collected for the two electrode systems. This proves that the device can operate up to a wide voltage of 2 V. Both the rGO and nanoparticles of the copper anchored boron doped graphene nanoplatelets are pseudo capacitors with a high specific capacitance. The properties of our asymmetric supercapacitor can be ascribed to the individual properties of the positive and negative electrode and their combination.

The rectangular curve of the CV shows the retention of the capacitance at different scan rates without the loss of the CV curve, irrespective of the potential windows from 0–2 V at 50 mV s^−1^ is shown in Fig. 7d. At the highest cell voltage of 2.0 V, the ASSC exhibits a redox-peak and a substantial rectangular shaped CV, which designates the co-operation of EDLC and the pseudocapacitance. Compared with the symmetric device, the redox peak at ASSC was lower. At a low scan rate of 10 mV s^−1^, 180 Fg^−1^ of specific capacitance was obtained, even at a high scan rate of 100 mV s^−1^ 128 Fg^−1^ of specific capacitance was still retained.

**Fig. 8:**
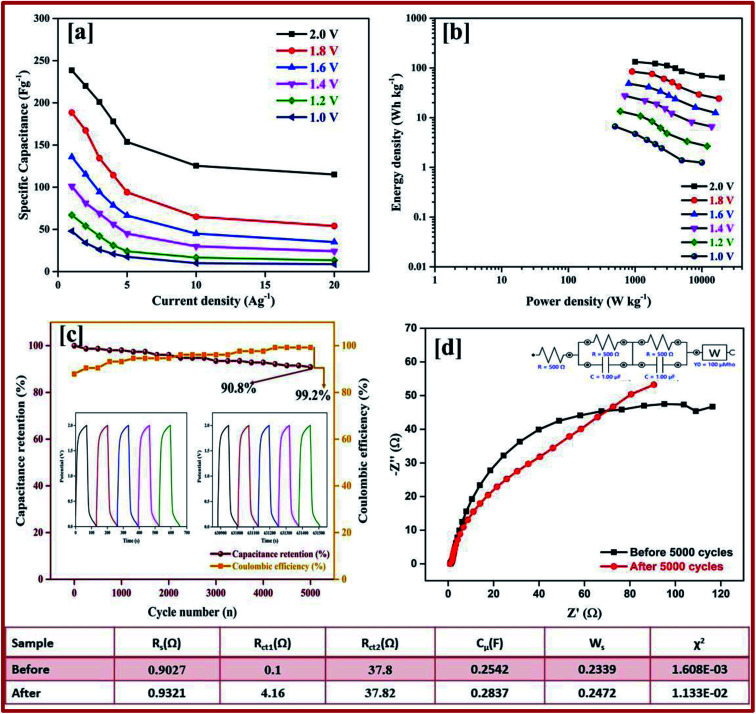
(a) Specific capacitance at different potentials and at different current densities from 1 to 20 Ag^−1^ calculated from the discharge curve. (b) Energy and power density calculated from the specific capacitance at different potentials and at different current densities. (c) Percentage of capacitance retention and percentage of coulombic efficiency of rGO//CuBG4 for 5000 cycles. (d) Nyquist plot before and after the cyclic stability test of 5000 cycles, the inset shows the circuit used for the calculation and the table showing the electrochemical studies is shown.

Galvanostatic charge–discharge measurement play a vital role in the asymmetrical soli-state supercapacitor to calculate the specific capacitance at different current densities and rate capabilities up to 5000 cycles. In various current densities the GCD of the device was evaluated (Fig. S10†). In Fig. 7e, the charge–discharge of the device at different potentials is shown at 0.5 Ag^−1^, this shows a regular charge and discharge at various potentials of 0.8–2.0 V. The specific capacitance of the device at different potentials was calculated at a current density of 0.5 Ag^−1^ and plotted in Fig. 7f. In a high potential window of 2.0 V, from the slope of the discharge curve the specific capacitance was 238.5 Fg^−1^ at a current density of 1.0 Ag^−1^. The specific capacitance of 125 Fg^−1^ was obtained at a high current density of 20 Ag^−1^. Even at a high current density the specific capacitance was almost 52.5%, this highlights that even at a high current density the device remains at maximum capacitance. The insertion of the first and last cycle in the cyclic stability also establish the same result. Interestingly, the charge–discharge of the supercapacitor and our asymmetric supercapacitor device were similar only at a low current density of 0.5 Ag^−1^. In Fig. 8c at a current density of 5 Ag^−1^, the cyclic stability of the asymmetric supercapacitor was measured up to 5000 cycles, and even after 5000 cycles 91% of the capacitance was retained without destruction of the charge–discharge. During the stability process the coulombic efficiency was close to 100%. Fig. 8a shows the specific capacitance of the device at different potentials of 0.8, 1.0, 1.2, 1.4, 1.6, 1.8 and 2 V and at different current densities (from 1 to 20 Ag^−1^), from the graph it can be seen that the capacitance is stable up to a high current density.

Electrical impedance studies were carried out for all of asymmetric supercapacitor devices in the frequency range of 100 kHz to 0.01 Hz. The fast ion transport between the positive and negative electrode was confirmed using a Nyquist plot as shown in Fig. 8d. The *R*_s_ equivalent series resistance represents the intercept of the first point on the real axis *Z*′ (Ω). In contrast, both the spectra show a straight line in the low frequency region, which indicates the excellent supercapacitor behaviour of the electrode. In the high frequency region an arc shape indicates the high stability in the electrochemical reaction. From the spectra, even after 5000 cycles, the value of *R*_s_ is almost unchanged and a slight increase in the *R*_ct_ value is obtained.

The high performance of the fabricated device was evaluated using a Ragone plot, which determines the relationship between the energy density (*E*) and power density (*P*). The energy density of rGO//CuBG4 (132.5 W h kg^−1^) was much higher than the other asymmetric devices of rGO//CuBG5 (112.2 W h kg^−1^) and rGO//CuBG3 (101.3 W h kg^−1^) at a same power density of 1000 W kg^−1^ in a cell voltage of 2.0 V. Significantly, without forfeiting the power density the ASSC renders a high energy density. The energy density of rGO//CuBG4 was 132.5 W h kg^−1^ at a current density of 1.0 Ag^−1^, even when the power density increased to 20 000 W kg^−1^, the energy density was maintained at up to 63.88 W h kg^−1^ at a current density of 20 Ag^−1^. The maximum energy density obtained for rGO//CuBG4 was 132.5 W h kg^−1^ at a current density of 1 Ag^−1^, which is much higher than that reported for other asymmetric supercapacitors (Table S1†). Fig. 8b shows the energy and power density of the rGO//CuBG4 for different potentials at different current densities, this shows that the energy and power density remains constant with the increase from a low potential to a high potential without any disturbance in the charge–discharge. Similarly, the other two devices of rGO//CuBG3 (Fig. S4–S6†) and rGO//CuBG5 (Fig. S7–S9†) are also given in the ESI† for further comparison. The electrochemical function of the fabricated solid-state asymmetric supercapacitor can be assigned by the combination of highly crystalline nanoparticles of copper anchored on a boron doped graphene nanosheet with rGO electrodes, the cells are fabricated using the gel electrolyte containing a polymer and H_2_SO_4_ to give a fast ion transfer and to reduce the cost of the device.

### Electrochemical performance of CuBG electrode in KOH

#### Positive electrode material

Cyclic voltammetry, GCD and EIS were carried out to evaluate the electrochemical performance of the electrode materials. To obtain a higher capacitance, 6 M KOH was used as the electrolyte. The entire work was carried out in the three-electrode system as mentioned above.

**Fig. 9:**
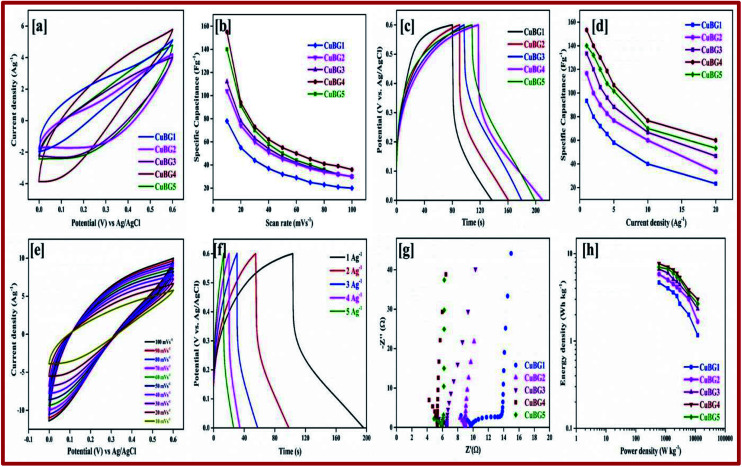
(a) CV of all CuBG samples at a scan rate of 10 mV s^−1^. (b) Variation of the specific capacitance of CuBG samples at different scan rates. (c) Charge/discharge curve of CuBG samples at 1 Ag^−1^. (d) Specific capacitance of samples at different current densities from 1 to 20 Ag^−1^. (e) CV of CuBG4 at different scan rates from 100 to 10 mV s^−1^. (f) Charge/discharge curve for CuBG4 at different current densities from 1 to 20 Ag^−1^. (g) EIS-Nyquist plot for all of the CuBG samples. (h) Energy and power density of all samples calculated from the discharge time at different current densities in the KOH electrolyte.

The difference between the faradic and non-faradic reactions was estimated using CV. Fig. 9a presents the CV curves for CuBG1, CuBG2, CuBG3, CuBG4 and CuBG5 in a positive potential range of 0–0.6 V (*vs.* Ag/AgCl) at a similar scan rate of 10 mV s^−1^. All of the CV curves reveal EDLC behavior. Fig. S11† shows the CV and charge discharge curves for CuBG1, CuBG2, CuBG3 and CuBG5. The specific capacitance was calculated from the CV curve and is presented in Fig. 9b for all of samples at different scan rates, and shows the gradual decrease in the capacitance and the increase in the scan rate without showing any sudden disturbance in the capacitance.

The areas of the curves are in the order CuBG4 > CuBG5 > CuBG3 > CuBG2 > CuBG1, that is, copper anchored onto a boron doped graphene nanosheet at a temperature of 850 °C has a high area value, which was further confirmed by GCD. Fig. 9e shows the CV curve of CuBG4 at various scan rates of 100–10 mV s^−1^ in the range of 0–0.6 V.

Owing to the high electrolyte concentration no sharp anodic and cathodic peak appears in the electrode materials. In the three electrode system, the galvanostatic charge/discharge was performed in a potential range of 0–0.6 V at a current density between 1 to 20 Ag^−1^.

The pseudocapacitive behavior of the electrode materials are indicated by the contribution of the double layer. Fig. 9c shows the charge/discharge for all of the samples at 1 Ag^−1^, from the discharge curve the capacitance was calculated, which confirms CuBG4 (153 Fg^−1^) is higher than the other materials CuBG5 (140 Fg^−1^) > CuBG3 (133 F g^−1^) > CuBG2 (116 Fg^−1^) > CuBG1 (93 F g^−1^). The specific capacitance of all of the samples at different current densities 1 to 20 Ag^−1^ are given in Fig. 9d, which proves that even at high current densities the capacitance is retained at 40% without loss of distortion in the shape of the charge/discharge.

The EIS of all of the samples are given in Fig. 9g, which shows a small semi-circle which intercepts at the real axis with a small resistance of less than 2 at higher frequency, and a steep increase of almost 90° in a low frequency region, implying a high ionic conductivity between the electrode materials and the electrolyte interfaces. From the spectra it can be seen that CuBG4 has a very small resistance (*R*_ct_) compared with the other materials. Thus, CuBG4 shows a high conductivity compared to the others in the KOH electrolyte as well shown in Table 2.

**Table 2:** Electrochemical parameters for the EIS-Nyquist plot in KOH

Sample	*R*_s_ (Ω)	*R*_ct1_ (Ω)	*R*_ct2_ (Ω)	*C*_μ_ (F)	*W*_s_	χ^2^
CuBG1	1.748	9.434	36.75	0.186	0.4398	8.62 × 10^−4^
CuBG2	1.752	6.934	29.68	0.305	0.1009	1.21 × 10^−4^
fCuBG3	1.0001	4.313	20.53	0.261	0.1857	4.53 × 10^−4^
CuBG4	0.2266	1.801	48.97	0.861	0.0459	2.16 × 10^−6^
CuBG5	0.3023	2.927	15.19	0.145	0.0277	1.52 × 10^−6^

The performance of the individual materials was reported by the energy and power density in the Ragone plot, this was calculated by using the specific capacitance at different current densities, as given in Fig. 9h. Compared to the other samples, CuBG1 4.6 W h kg^−1^, CuBG2 5.8 W h kg^−1^, CuBG3 6.66 W h kg^−1^, and CuBG5 7 W h kg^−1^, CuBG4 exhibits a higher energy density of 7.66 W h kg^−1^ at 600 W kg^−1^ which is in the supercapacitor region.

#### Negative electrode material

For the ASSC with the KOH/PVA gel electrolyte, activated carbon was used as the cathode (negative) electrode material. The activated carbon performance was evaluated using the three-electrode system. Fig. S12a† shows the CV of the activated carbon at different scan rates from 100 to 10 mV s^−1^ in the potential range of −1 to 0 V. The rectangular shape of the CV curve shows the characteristics of EDLC in charge storage. Using the integrated value from the curve in eqn (1) the capacitance of the materials was calculated. Activated carbon shows 72 Fg^−1^ at a scan rate of 10 mV s^−1^, as we increase the scan rate from 10 to 100 mV s^−1^, there was a gradual decrease in the specific capacitance owing to the fast movement of the ions on the surface itself, without the passage of ions into the electrode material.

Fig. S12b† shows the charge/discharge of AC at the different current densities of 1, 2, 3, 4, 5, 10 and 20 Ag^−1^. From the discharge time, the specific capacitance was calculated using eqn (2). Fig. S12c† shows the specific capacitance of AC at different current densities.

#### Asymmetric supercapacitor of CuBG4 in KOH/PVA

**Fig. 10:**
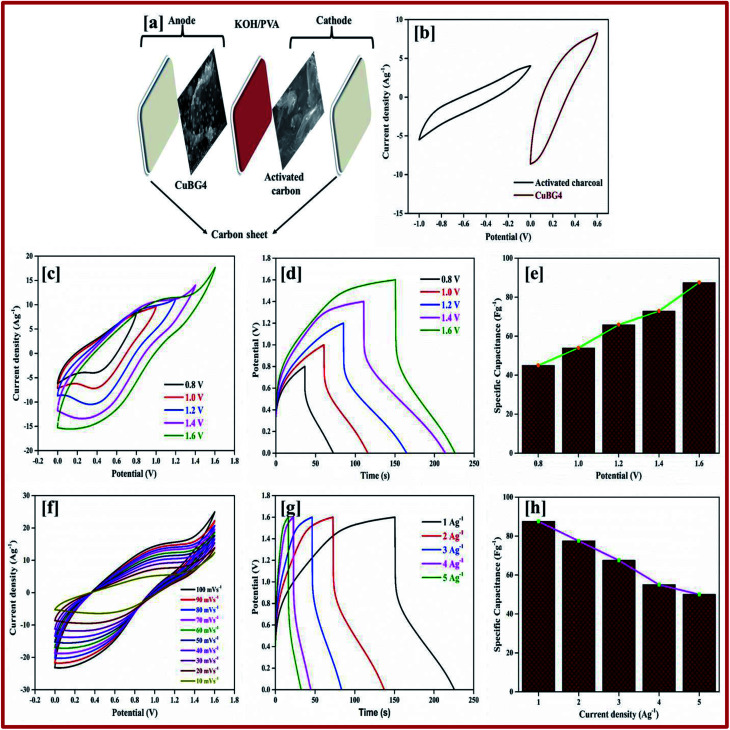
(a) Schematic diagram of CuBG4. (b) CV of CuBG4//AC device at 50 mV s^−1^. (c) CV curve of CuBG4 at different potentials from 0.8 to 1.6 V. (d) Charge–discharge curve of CuBG4 at different potentials. (e) Specific capacitance of CuBG4 at different potential windows. (f and g) CV and charge–discharge curve of CuBG4 at different scan rates in a high potential of 1.6 V; and (h) the specific capacitance of CuBG4 at different current densities from 1 to 5 Ag^−1^ in a potential of 1.6 V in a KOH/PVA electrolyte.

For the fabricated ASSC device of CuBG4//AC, the AC was used as a negative electrode and CuBG4 was used as a positive electrode in KOH/PVA gel electrolyte. The electrochemical properties of the electrodes were first estimated *via* CV in a three-electrode system with Pt as the counter electrode and Ag/AgCl as the reference electrode. The stable potential window of activated carbon between −1 to 0 V and CuBG between 0–0.6 V in the positive region were obtained. The sum of the potential range of the activated carbon (1 V) and CuBG (0.6 V) was extended up to 1.6 V in the KOH/PVA electrolyte solution for the ASSC of CuBG//AC (Fig. 10b). It is necessary to balance the charges in the positive and negative electrodes as mentioned above in eqn (8), the mass of each electrode was balanced to fabricate the ASSC device of CuBG//AC. Accordingly, the overall electrochemical performance was investigated in the 0–1.6 V potential window.

Fig. 10c shows the CV curve of the ASSC device at different potential window ranges from 0.8–1.6 V at a scan rate of 50 mV s^−1^, which shows a regular increase in the EDLC behavior with a distorted rectangular shape owing to the contribution of the faradic positive electrodes. At a high potential of 1.4–1.6 V a very small redox peak was observed owing to the insertion and extraction of the ions into the electrode materials during the oxidation and reduction process. However, as the potential increases to more than 1.7 V, decomposition of electrolyte is evident with the evolution of oxygen/hydrogen, which increases the current dramatically, whereas the stability of the material decreases.

The CV curves shown in Fig. 10f result in both regular EDLC and pseudocapacitive behaviors at a high potential window of 1.6 V with different scan rates (10 to 100 mV s^−1^). From eqn (1) the specific capacitance was calculated as 87 Fg^−1^ at a scan rate of 10 mV s^−1^, which is higher than the activated carbon electrode. This is owing to the overall combination of the primary pseudocapacitance and the redox property of the copper and boron, and the EDLC behavior of the graphene sheet in the device. Additionally, it was observed that both the materials are stable at a higher potential. The specific capacitance increases with a decrease in the scan rate from 100 to 10 mV s^−1^, as the movement of ions is limited at high scan rate only on the outer active surface, resulting in low utilization of the electroactive materials.

**Fig. 11:**
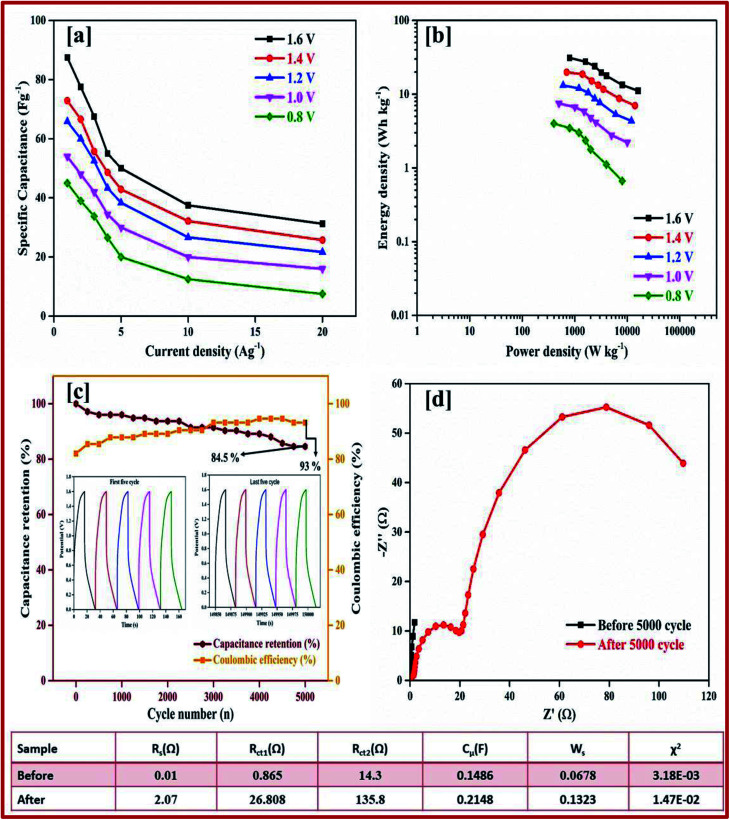
(a) Specific capacitance at different potentials and at different current densities from 1 to 20 Ag^−1^ calculated from the discharge curve. (b) Energy and power density calculated from the specific capacitance at different potentials and different current densities. (c) Percentage of capacitance retention and percentage of coulombic efficiency for CuBG4//AC for 5000 cycles in a KOH/PVA electrolyte. (d) Nyquist plot before and after the cyclic stability test of 5000 cycles.

The ASSC device was further evaluated using GCD at various current densities from 1 to 20 Ag^−1^. Fig. 10d shows the charge–discharge curve of the ASSC device at different potential ranges from 0.8–1.6 V at a current density of 1 Ag^−1^. This shows the regular increase in the discharge time(s). There is a non-linear curve in the charge–discharge curve at very low current densities owing to the redox reaction from the copper on the electrode materials and the results remain close to the CV curve. From the slope, the initial drop of voltages (*IR*) in the discharge curve for the internal resistance in the active materials were determined at different current densities. Fig. 10g shows the charge–discharge curve of the ASSC device at 1.6 V at different current densities from 1 to 5 Ag^−1^. From the discharge curve the capacitance was calculated and is given in Fig. 10h, this indicates the gradual decrease in the specific capacitance at higher current densities. Fig. 11a gives the specific capacitance at different current densities with different cell voltages from 0.8 to 1.6 V.

The energy and power density of the materials was calculated using the eqn (3) and (4). Fig. 11b shows the energy and power density at various current densities, it is evident that CuBG4 has the highest energy density of 31.1 W h kg^−1^ and a power density of 800 W kg^−1^ at 1 Ag^−1^ in a cell voltage of 1.6 V. The cyclic stability test for CuBG//AC was carried out over 5000 cycle at 5 Ag^−1^, in the highest potential range of 0 to 1.6 V. Fig. 11c, and shows the capacitance retention of the ASSC device at different cycle numbers. The ASSC device exhibits an excellent electrochemical stability with a deterioration of 15% of the specific capacitance from the initial capacitance after 5000 cycles. This is due to the inactive boron in the active materials on the KOH/PVA solid electrolyte, as it is active on the H_2_SO_4_/PVA electrolyte. Hence, the electroactive materials show a very high stability in the H_2_SO_4_/PVA electrolyte compared to the KOH/PVA solid electrolyte.

The fundamental behavior of supercapacitor was examined using EIS analysis. Furthermore, the impedance of the ASSC device was measured after the 1^st^ and 5000^th^ cycle in the frequency range of 1 MHz to 0.1 Hz at an open circuit potential with an AC perturbation of 2 mV. The obtained impedance is similar to an arc at a high frequency and a spike at a lower frequency, which demonstrates the long-term stability of this ASSC device. The impedance spectra were analyzed using the Z-Simp win fitting method on the basis of an equivalent circuit and is shown in the inset of Fig. 11d. At a very high frequency, the combined resistance of the electrolyte and the ions and contact resistance of active materials (*R*_s_) intercept at a real part (*Z*). The *R*_s_ value is almost same for both of the spectra. The semicircle at a low frequency shows a major difference owing to faradic reaction and the double-layer on the surface, which corresponds to the charge-transfer resistance (*R*_ct_). Owing to the Warburg resistance (*Z*_w_) the slope of the curve was obtained, which is the ion diffusion to the electrode surface.

After 5000 cycles the charge-transfer resistance was increased from 0.8 to 6.8 owing to the corrosion caused by the dissolved oxygen in the electrolytes on the current collector during the charge–discharge cycle. Owing to the high concentration of electrolytes there was not a significant increase in the *R*_ct_ value before and after 5000 cycles.

## Conclusion

In this study, a novel and facile hydrothermal process, followed by a CVD method was utilized for the synthesis of copper anchored boron doped graphene nanoplatelets as active electrode materials. We designed a long-lasting and indestructible ASSC employing prepared electrode materials. In the solid-state symmetric supercapacitor CuBG4 shows a high capacitance of 483.3 Fg^−1^ at a low current density of 1 Ag^−1^ and demonstrated a high cyclic stability even at a current density of 5 Ag^−1^, It also showed a capacitance retention of 91%, even after 5000 cycles. The symmetric solid-state supercapacitor exhibits a high energy and power density at 1 Ag^−1^. The novel ASSC device utilized rGO as the positive electrode and CuBG as the negative electrode with H_2_SO_4_/PVA as the solid electrolyte, which was calibrated at a high potential window of 2.0 V. At a current density of 1 Ag^−1^ the device exhibits an eminent energy density of 132.5 W h kg^−1^ at a power density of 1000 W kg^−1^, respectively. The device exhibits an excellent cyclic stability, even after 5000 cycles with acapacitance retention of 90.8%. We made a single asymmetric device utilizing the CuBG material as a positive electrode and activated carbon as the negative electrode material with KOH/PVA as the solid electrolyte and tested green LEDs (2 V) to determine the long life stability. The device showed a high energy density of 31.1 W h kg^−1^ at a power density of 800 W kg^−1^ in a potential window of 1.6 V. At a current density of 5 Ag^−1^ it shows a high cycle stability with an 84.5% capacitance retention and a 93% coulombic efficiency.

## Conflicts of interest

The authors declare no conflict of interest.

## Supplementary Material

RA-009-C8RA08762H-s001
