# Supplementary material for: Copper nanoparticles anchored onto boron-doped graphene nanosheets for use as a high performance asymmetric solid-state supercapacitor
Source: RSC Adv. 2019 Jan 25;9(6):3443–61. doi: 10.1039/c8ra08762h (PMC9088218; doi:10.1039/c8ra08762h)
Supplement: RA-009-C8RA08762H-s001 [file RA-009-C8RA08762H-s001.pdf]

Electronic Supplementary Material (ESI) for RSC Advances.
This journal is © The Royal Society of Chemistry 2019

Supporting Information

Copper Nanoparticles Anchored Boron Doped Graphene Nanosheet for High performance Asymmetric Solid-State Supercapacitors

P. Muthu Pandian and A. Pandurangan *

Department of Chemistry, Anna University, Chennai – 600 025, Tamil Nadu, India

*Correspondence: pandurangan_a@yahoo.com; Tel.: +91 44 22358653; fax: +91 44 22200660

Figure S1. Deconvoluted XPS spectra of O1s, (a) CuBG3 and (b) CuBG4

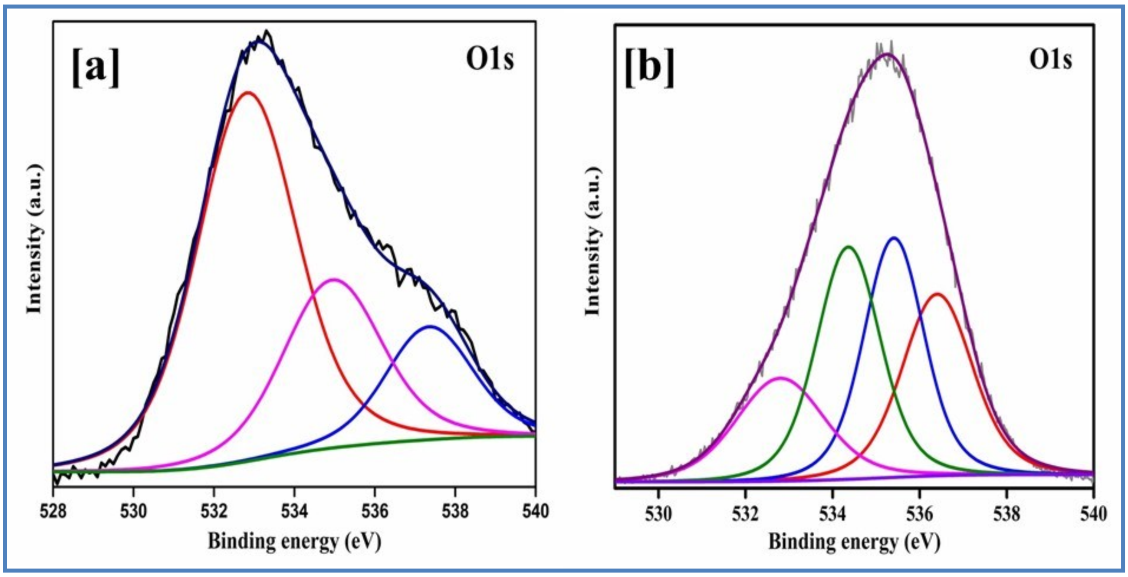

Figure S2. Cyclic voltammetry at different scan rate and Charge-discharge curve at different current density of CuBG5(a,b), CuBG3(c,d), CuBG2(e,f), CuBG1(g.h) in H_2SO_4/PVA

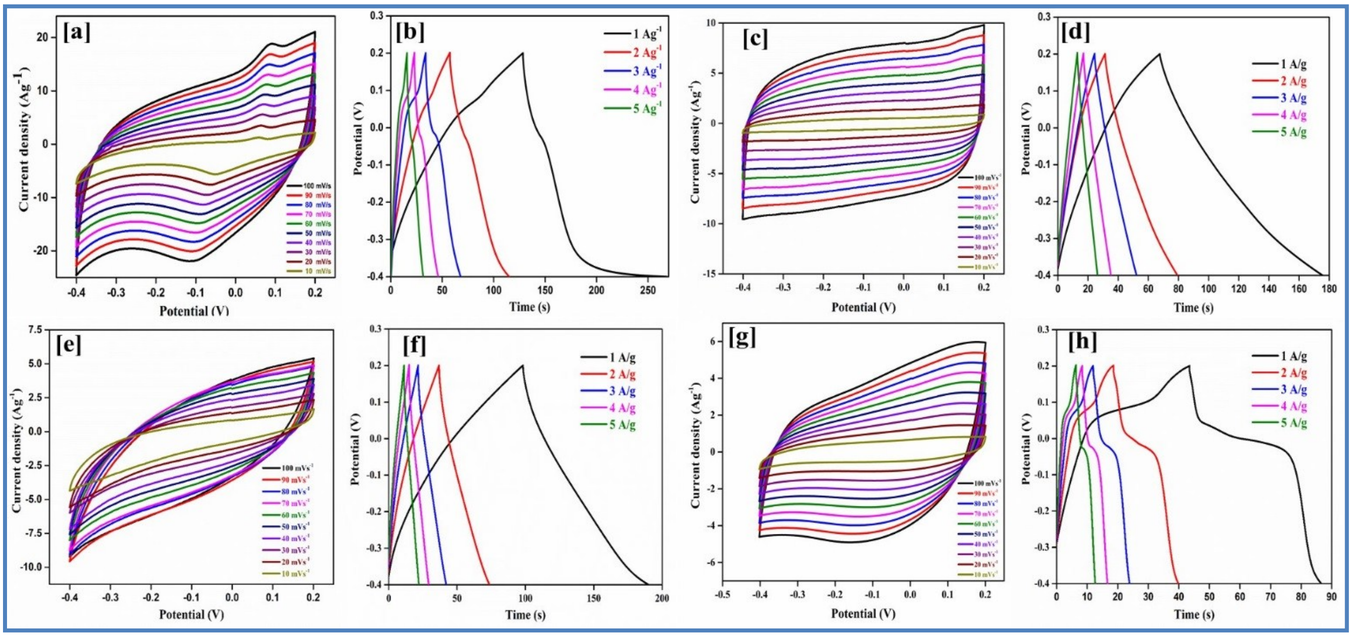

Figure S3. Capacitance retention and Coulombic efficiency of CuBG1(a), CuBG2(b), CuBG3(c), CuBG4(d) and CuBG5(e) with first and last five cycle

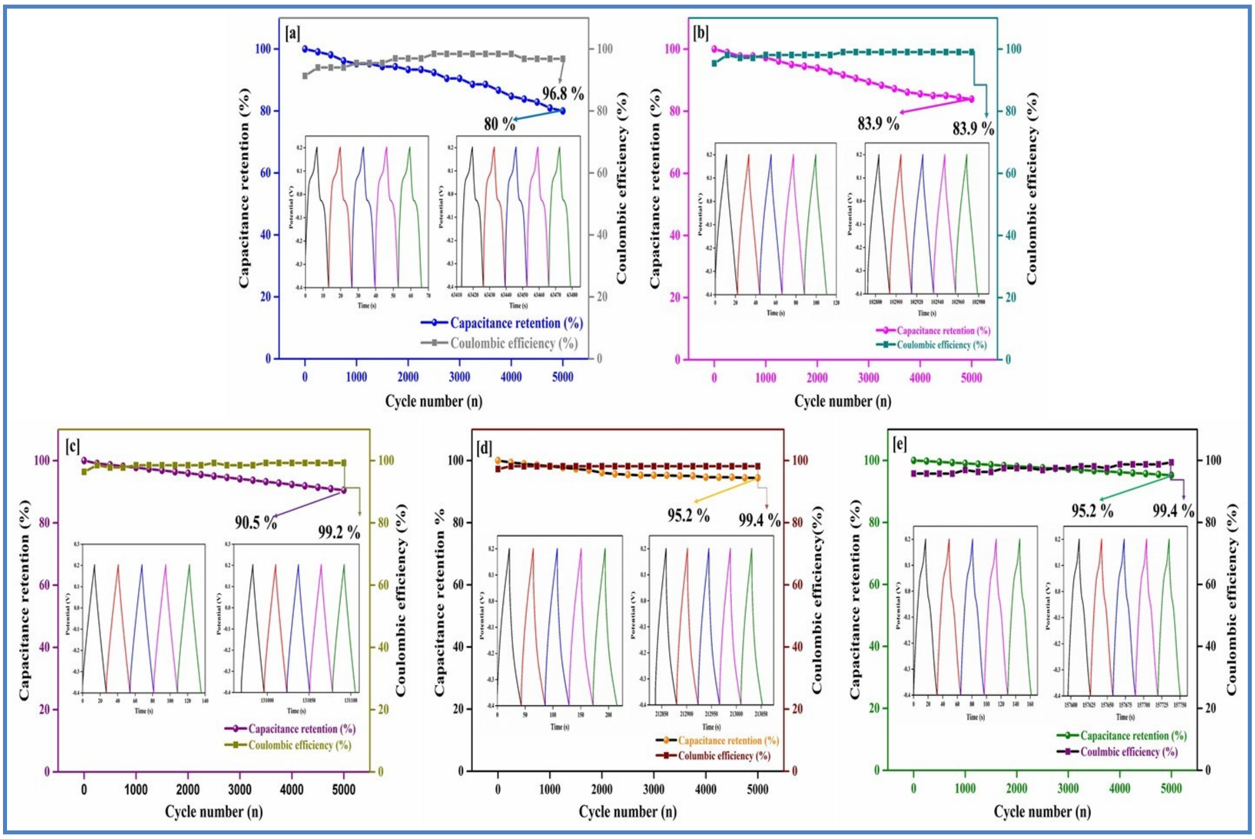

Figure S4. Graphical abstract of CuBG3 (a), Cyclic voltammetry of rGO//CuBG3 device at 50 mVs^{-1} (b), CV curve of CuBG3 at different potential from 0.8 V - 2.0 V (c), Charge-discharge curve of CuBG3 at different potential (d), Specific Capacitance of CuBG3 at different potential window (e), CV and charge-discharge curve of CuBG3 at different scan rate in a high potential 2.0 V (f,g) and the specific capacitance of CuBG3 at different current density (from 1 Ag^{-1} to 20 Ag^{-1}) in potential 2.0 V (h).

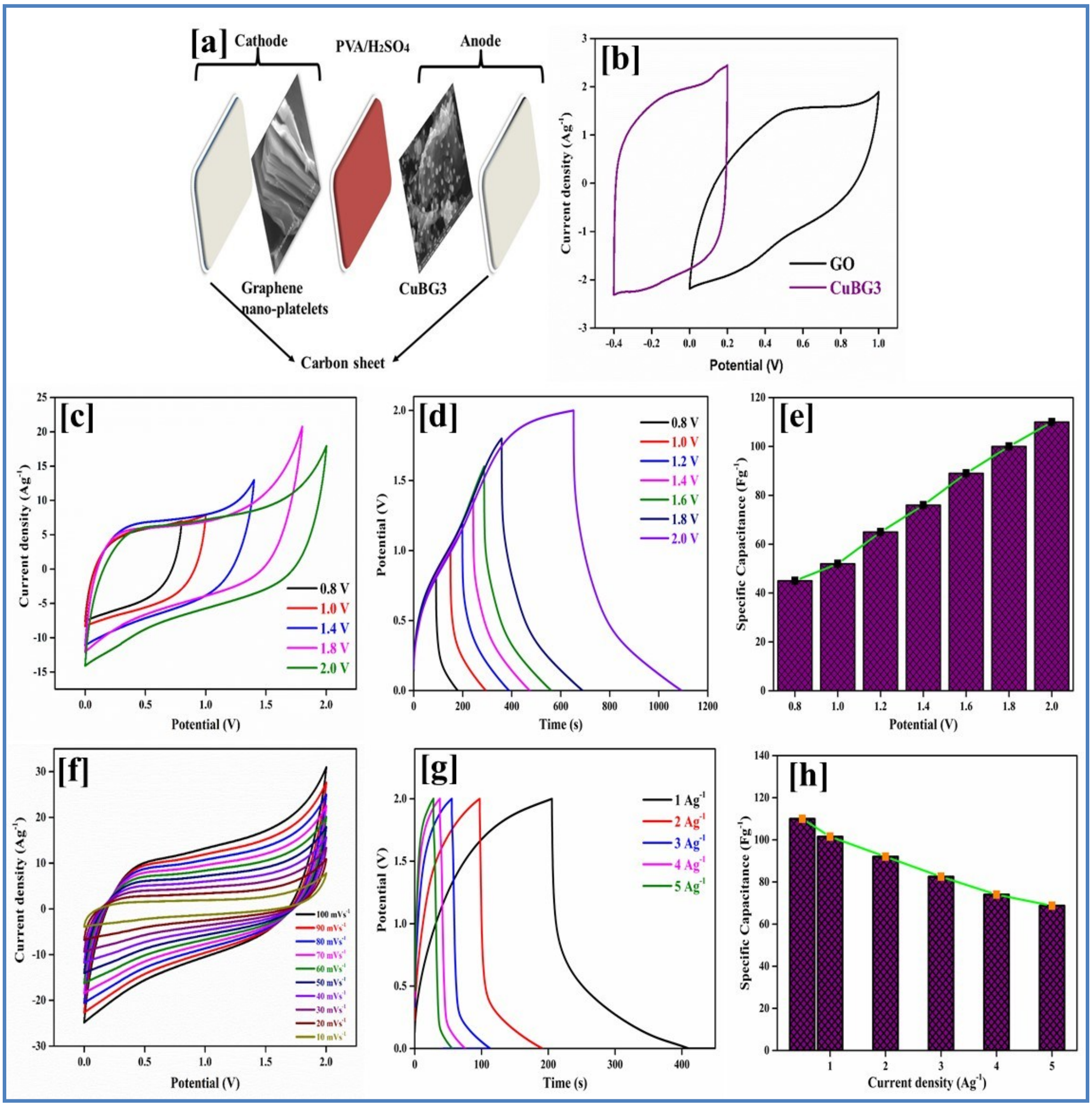

Figure S5. Cyclic voltammetry and charge-discharge curve of rGO//CuBG3 at different potential window

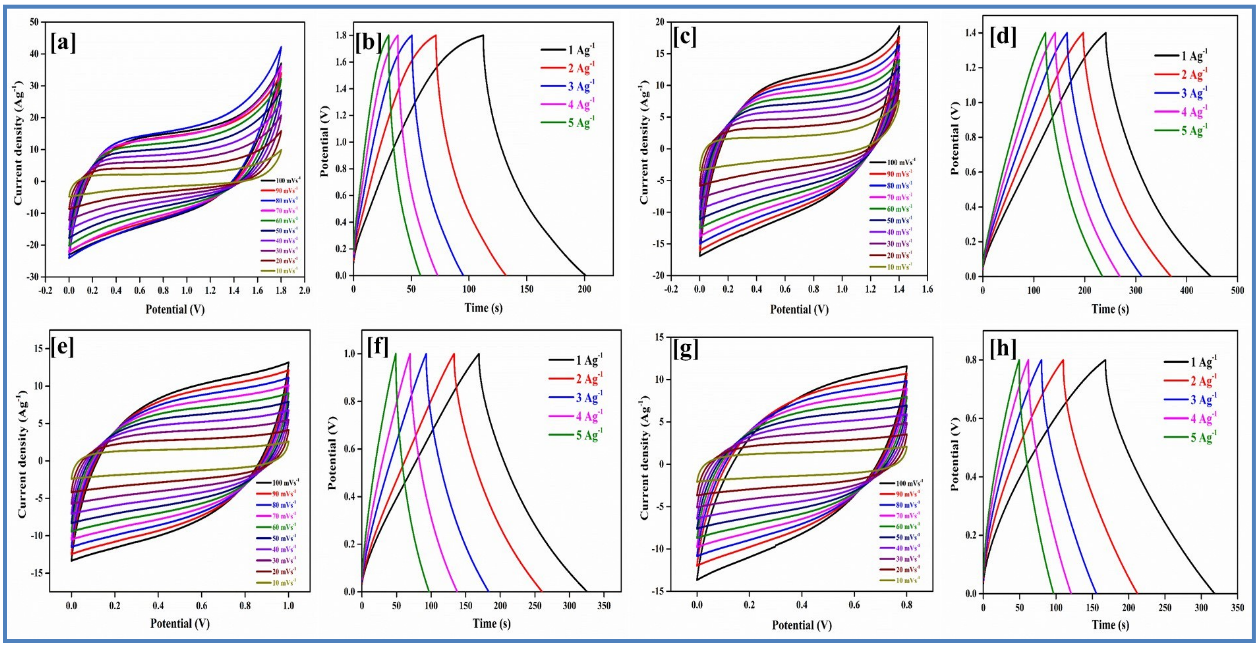

Figure S6. Specific capacitance at different potential and at different current densities from 1 Ag^{-1} to 20 Ag^{-1} calculated from the discharge curve(a), energy and power density calculated from specific capacitance at different potential and different current densities(b), % of capacitance retention and % of coulombic efficiency of rGO//CuBG3 for 5000 cycles(c), Nyquist plot before and after the cyclic stability of 5000 cycles(d) the circuit used for the calculation is inserted and the table with electrochemical studies are placed.

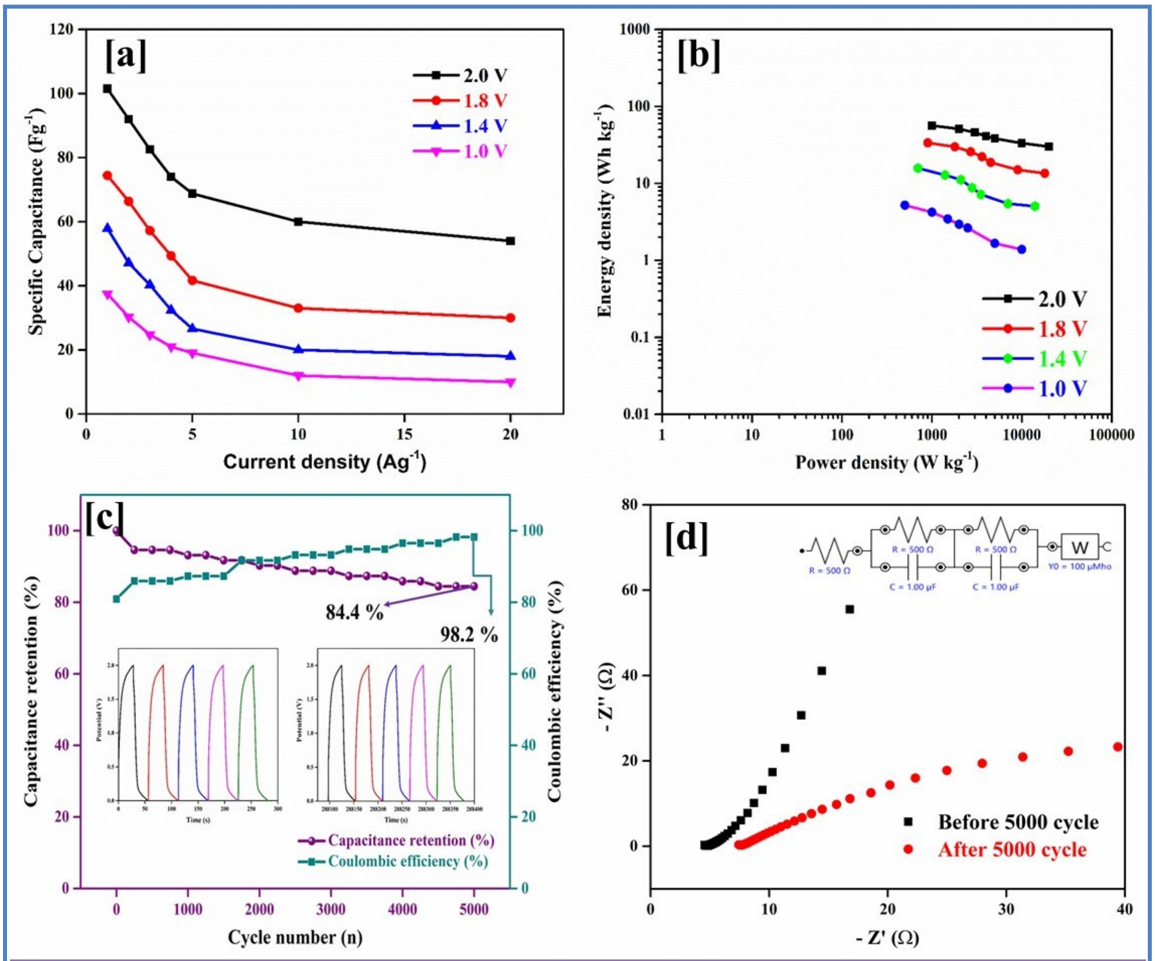

Sample	$R_s(\Omega)$	$R_{ct1}(\Omega)$	$R_{ct2}(\Omega)$	$C_\mu(F)$	W_s	χ^2
Before	4.168	0.5486	40.11	0.2622	0.2158	1.44E-04
After	7.691	1.621	55.46	1.958	0.2822	7.24E-03

Figure S7. Graphical abstract of CuBG5 (a), Cyclic voltammetry of rGO//CuBG5 device at 50 mVs^{-1} (b), CV curve of CuBG5 at different potential from 0.8 V - 2.0 V (c), Charge-discharge curve of CuBG5 at different potential (d), Specific Capacitance of CuBG5 at different potential window (e), CV and charge-discharge curve of CuBG5 at different scan rate in a high potential 2.0 V (f,g) and the specific capacitance of CuBG5 at different current density (from 1 Ag^{-1} to 20 Ag^{-1}) in potential 2.0 V (h).

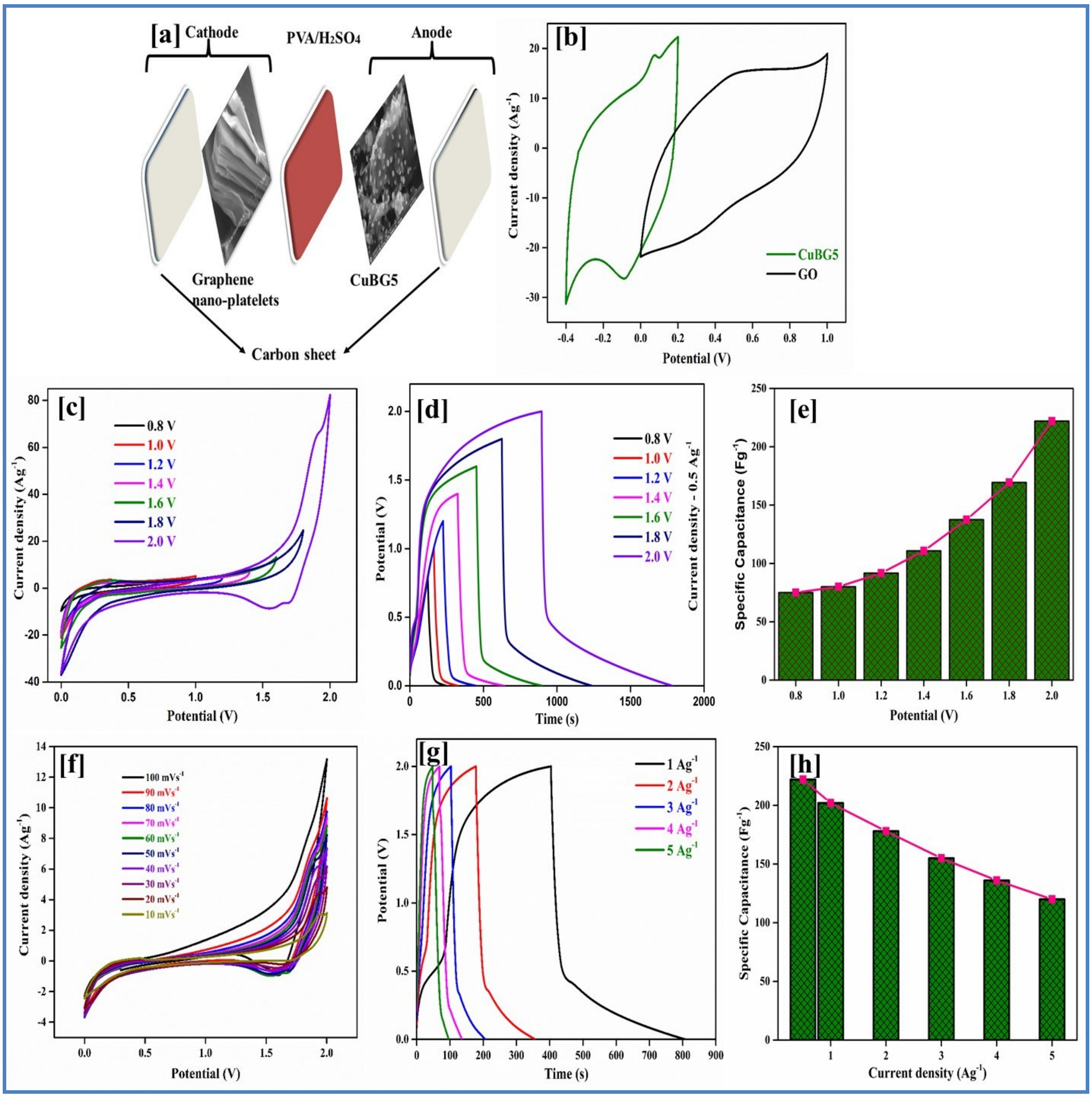

Figure S8. Cyclic voltammetry and charge-discharge curve of rGO//CuBG5 at different potential window

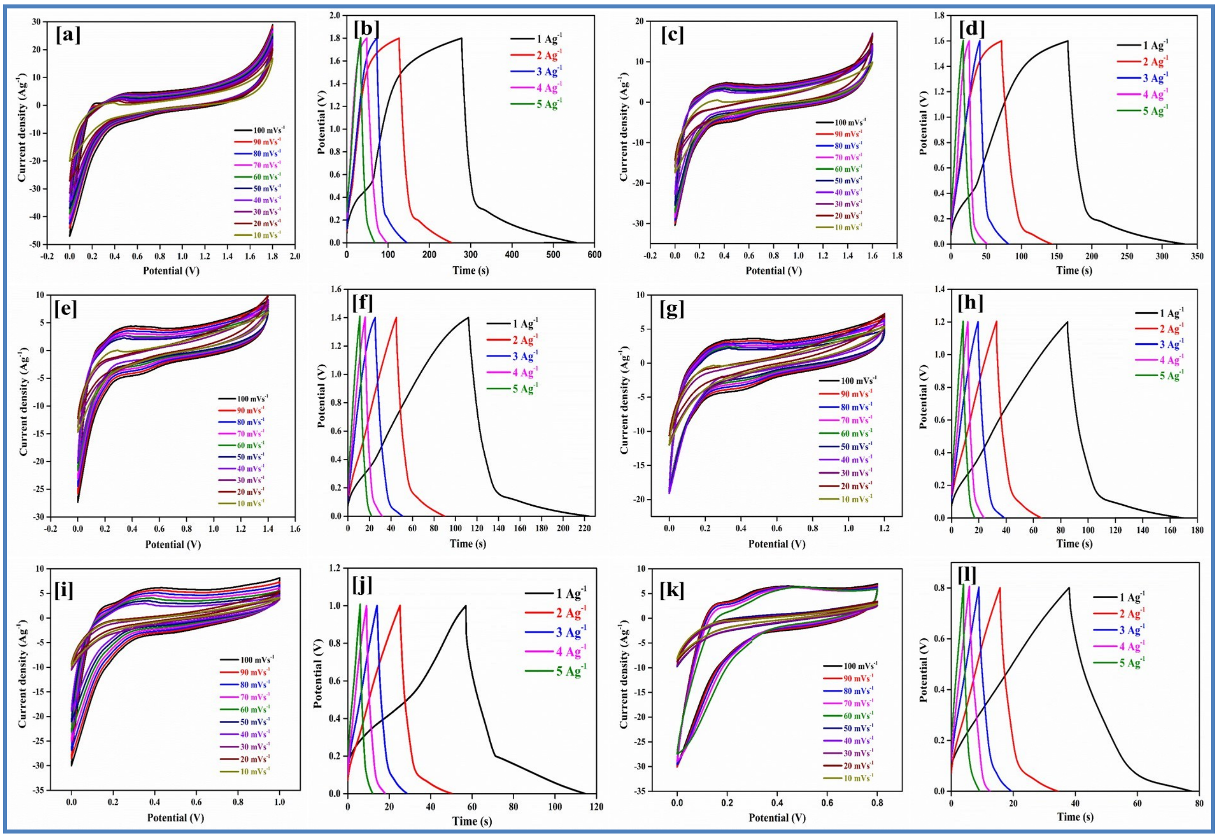

Figure S9. Specific capacitance at different potential and at different current densities from 1 Ag^{-1} to 20 Ag^{-1} calculated from the discharge curve(a), energy and power density calculated from specific capacitance at different potential and different current densities(b), % of capacitance retention and % of coulombic efficiency of rGO//CuBG5 for 5000 cycles(c), Nyquist plot before and after the cyclic stability of 5000 cycles(d) the circuit used for the calculation is inserted and the table with electrochemical studies are placed.

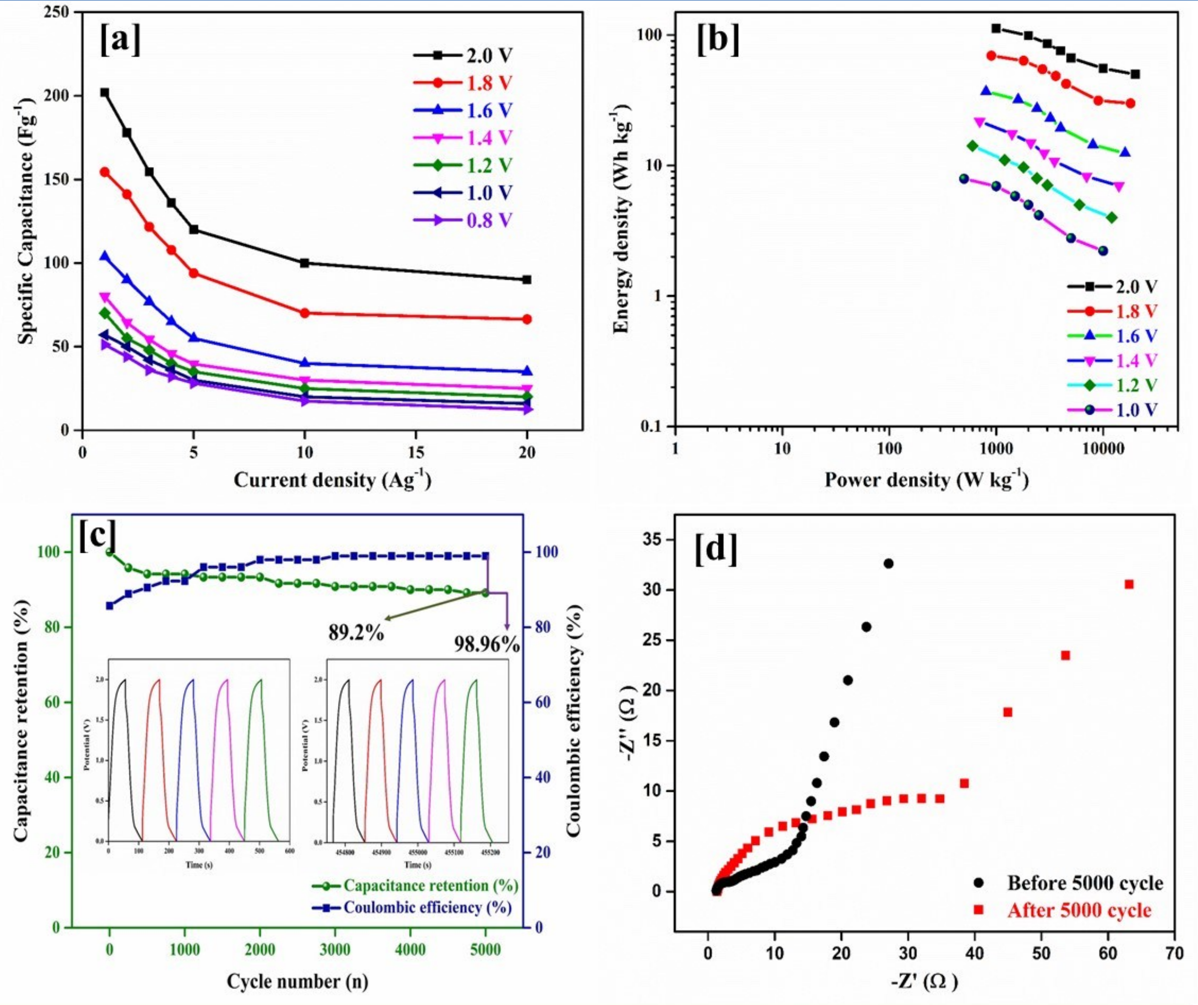

Sample	$R_s(\Omega)$	$R_{ct1}(\Omega)$	$R_{ct2}(\Omega)$	$C_\mu(F)$	W_s	χ^2
**Before**	1.277	0.2831	1.714	0.0044	0.3742	1.230E-02
**After**	1.295	0.2145	12.97	0.1957	0.295	1.397E-02

Figure S10. Cyclic voltammetry and charge-discharge curve of rGO//CuBG4 at different potential window in H_2SO_4/PVA

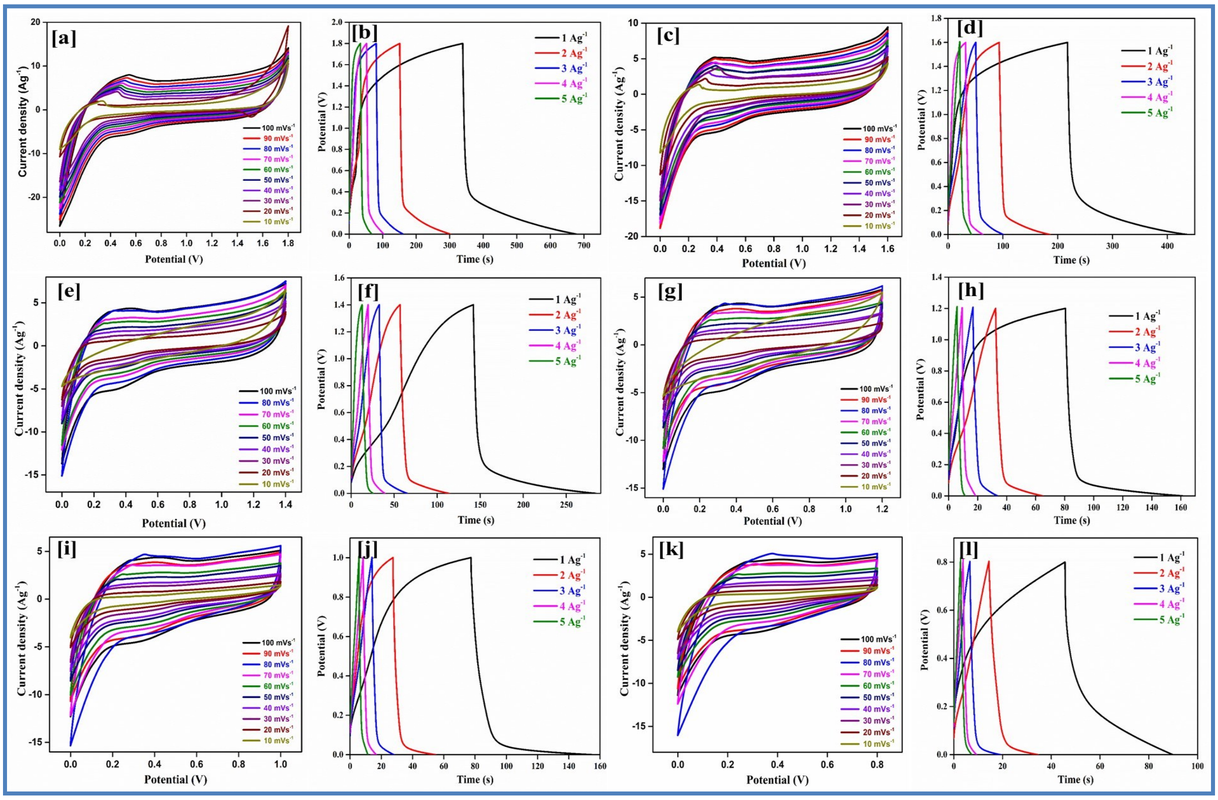

Figure S11. Cyclic voltammetry at different scan rate and Charge-discharge curve at different current density of CuBG1(a,b), CuBG2(c,d), CuBG3(e,f), CuBG5(g.h) in KOH

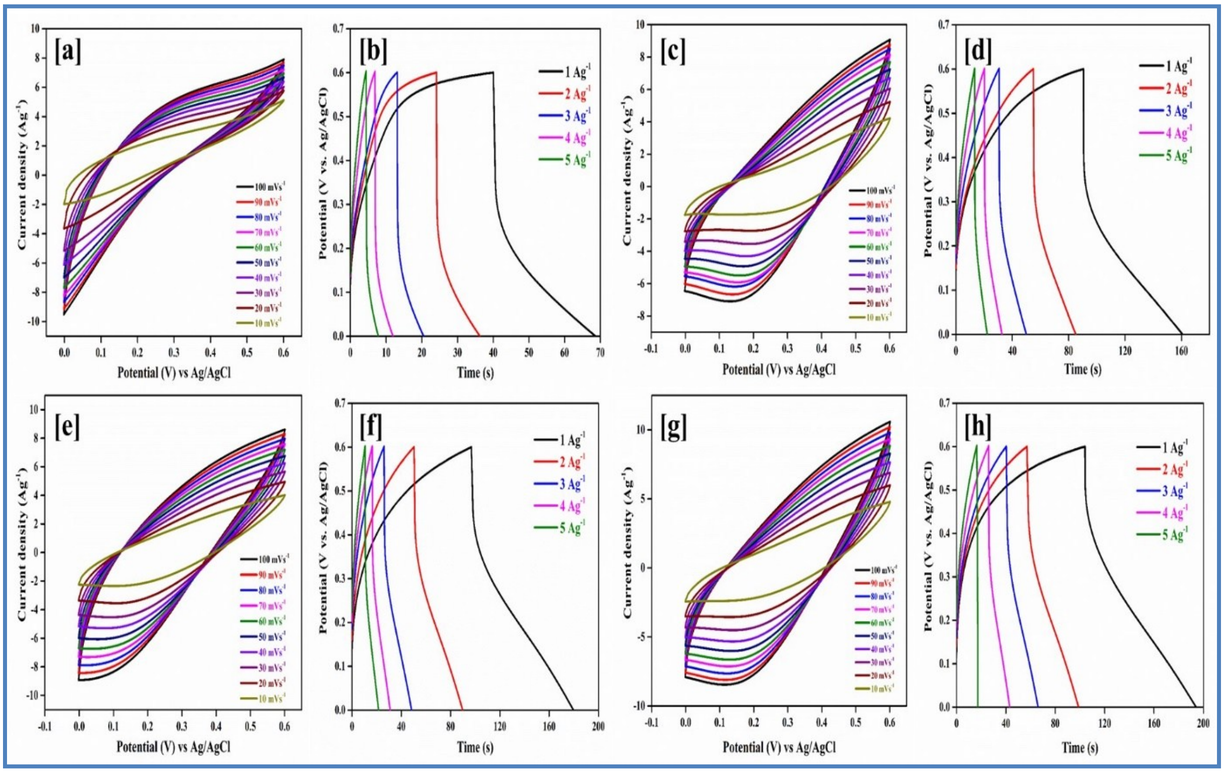

Figure S12. Cyclic voltammetry of activated charcoal at different scan rate, (b) Charge/discharge curve of AC at different current density and (c) Specific capacitance at different current density.

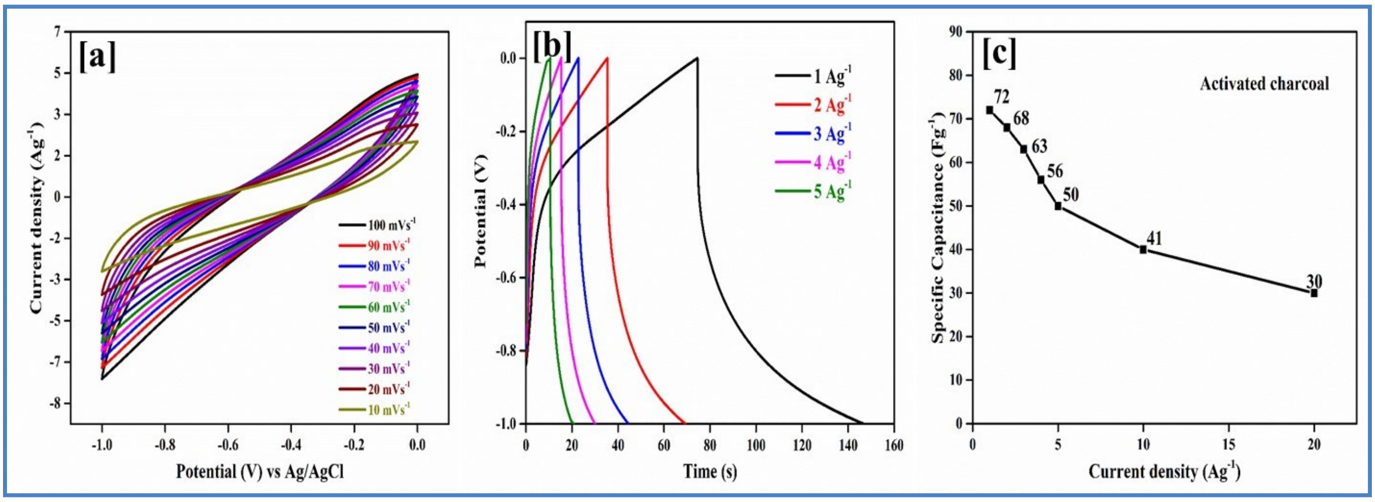

Figure S13. Cyclic voltammetry and charge-discharge curve of CuBG4//AC at different potential window in KOH/PVA

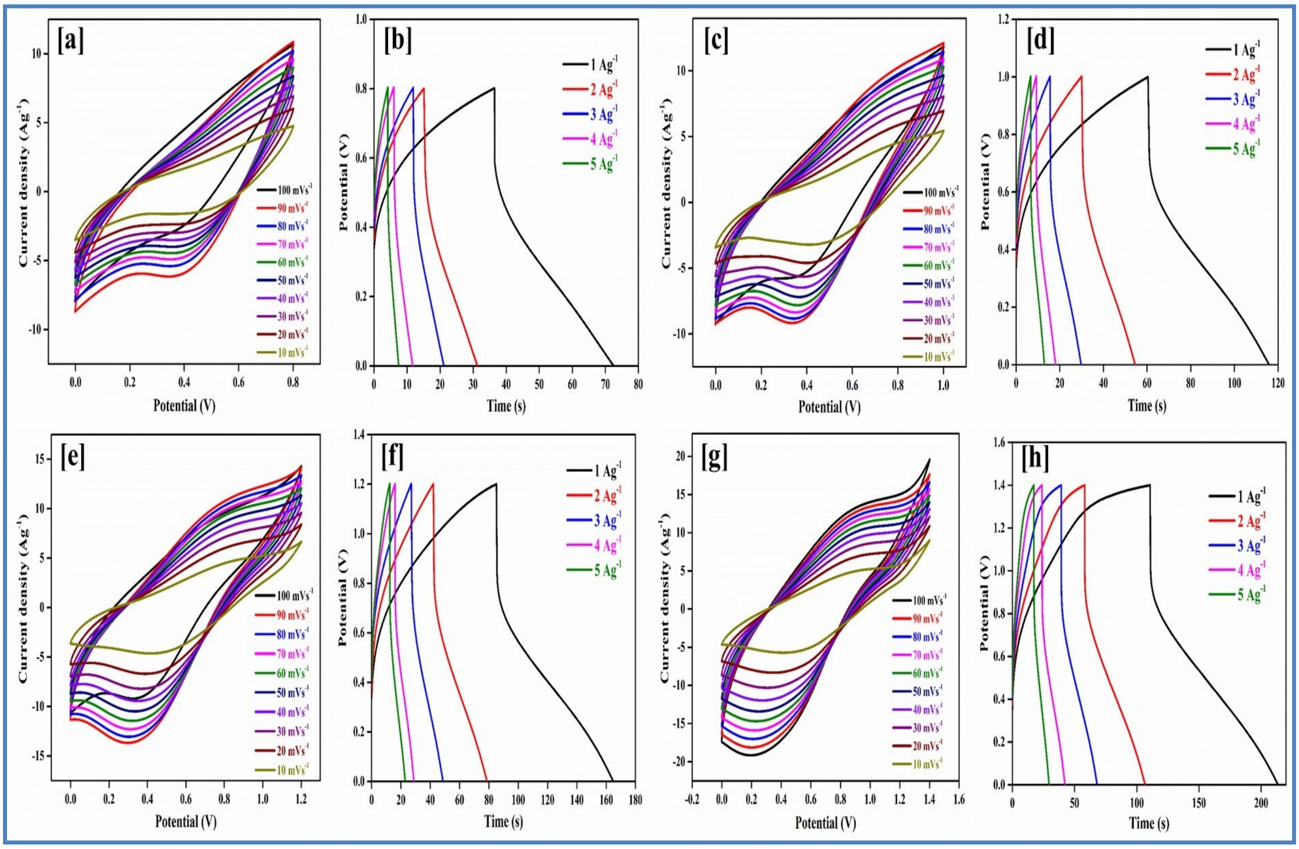

Table S1. Comparative study based on all solid-state asymmetric supercapacitor with different metal oxide/sulphide

S.No	Cathode	Anode	Cell Voltage (V)	Current density (Ag^{-1})	Csp (Fg^{-1})	Cycle life (No. of cycle)	Energy density (Wh kg^{-1})	Power density (W kg^{-1})	Reference
1	Co_3O_4	Carbon aerogel	1.5	1	57.4	85 % (1000)	17.9	750	1
2	MnS	Activated charcoal	1.6	1	110.4	89.87 % (5000)	37.6	181.2	2
3	Ni-Co-S	Graphene	1.8	2	133	82.2 % (20000)	60	1800	3
4	Fe_3O_4/ Carbon	Porous carbon	1.4	1	58.5	70.8 % (5000)	18.3	351	4
5	$Cu(OH)_2$	Activated charcoal	1.6	4	26.4	90 % (5000)	3.68	1253	5
6	Cu-B/Graphene nanosheet	Reduced Graphene oxide	2	5	153.75	90.84 % (5000)	85	5000	This work

References

1. W. Liu, X. Li, M. Zhu and X. He, *Journal of Power Sources*, 2015, **282**, 179-186.
2. T. Chen, Y. Tang, Y. Qiao, Z. Liu, W. Guo, J. Song, S. Mu, S. Yu, Y. Zhao and F. Gao, *Scientific Reports*, 2016, **6**, 23289.
3. J. Shi, X. Li, G. He, L. Zhang and M. Li, *Journal of Materials Chemistry A*, 2015, **3**, 20619-20626.
4. H. Fan, R. Niu, J. Duan, W. Liu and W. Shen, *ACS Applied Materials & Interfaces*, 2016, **8**, 19475-19483.
5. J. Chen, J. Xu, S. Zhou, N. Zhao and C.-P. Wong, *Journal of Materials Chemistry A*, 2015, **3**, 17385-17391.
